# Predicting Oncogenic Mutants of Epidermal Growth Factor Receptor in Lung Cancer by Molecular Dynamics Simulation with Machine Learning

**DOI:** 10.1021/acsomega.6c08155

**Published:** 2026-09-10

**Authors:** Victor O. Anyanwu, Justin Spiriti, Chung F. Wong

**Affiliations:** Department of Chemistry and Biochemistry, 14611 University of Missouri-St Louis, 1 University Boulevard, St. Louis, Missouri 63121, United States

## Abstract

The epidermal growth factor receptor (EGFR) is a frequently mutated protein kinase found in patients suffering from non-small cell lung cancer (NSCLC). Although therapeutic drugs targeting EGFR are available, not all patients respond to these drugs, and it is not yet known whether some clinically detected mutations are actionable. Although the oncogenicity of some mutants is well established, many mutants found in patients are not yet classified or classified as Variants of Uncertain Significance. For newly discovered rare mutants, strong evidence supporting their classification into oncogenic or benign is often lacking. Under such situations, multiple pieces of evidence can still help classification, even if each individual piece of evidence is weaker. Computational predictions have been considered as supporting information by the Standard Operating Procedure (SOP) of the Clinical Genome Resource/Cancer Genomics Consortium/Variant Interpretation for Cancer Consortium (ClinGen/CGC/VICC). The SOP considers predictions from multiple computational programs as providing only one piece of supporting information to avoid double counting because these programs share common approaches. A computational approach that explicitly accounts for the conformational effects of mutations via molecular dynamics (MD) simulation could add another piece of complementary supporting information. We tested the hypothesis that features of structural dynamics observed from MD simulation could help distinguish oncogenic from benign mutants. To this end, we performed three independent MD simulations lasting 1 μs each for wild-type EGFR and 32 of its oncogenic or benign mutants. We found that random forest machine-learning models constructed using suitable structural features were able to distinguish oncogenic and benign mutations effectively. Although we did not supply features of structural motifs commonly used by scientists to distinguish active and inactive conformations, our machine-learning approach identified and utilized some features associated with these motifs for classifying EGFR mutants.

## Graphical abstract

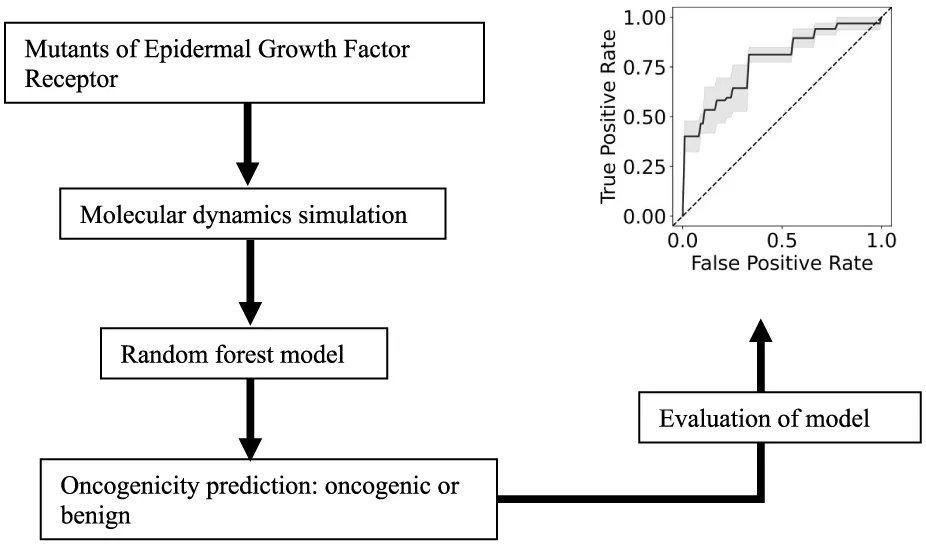

## Introduction

Lung cancer accounts for the most cancer deaths, partly because it is usually diagnosed at a late stage.^1^ Nevertheless, the death rate has decreased substantially in recent years. Discouraging smoking partly accounts for the decrease, as do new treatment options. Targeted therapy using protein kinase inhibitors (PKIs) represents one major advance in treatment. In 2012, the National Comprehensive Cancer Network (NCCN) recommended testing patients for genetic alternations of protein kinases that PKIs could target.^2^ In 2013, inhibitors for the epidermal growth factor receptor (EGFR) were further approved for first-line treatment of non-small cell lung cancer (NSCLC),^3,4^ which accounts for ∼75% of lung cancer in the US. In a recent population-based analysis,^5^ the decline in the death rate of NSCLC was found to be faster than the decline in the incidence rate after PKIs have been used liberally to treat patients. Two-year survival for men diagnosed with NSCLC in 2001, before PKIs were available for treating lung cancer, was only 26%. This improved to 35% for those diagnosed in 2014. The corresponding data for women were from 35% to 44%. As immune check point inhibitors were not yet in widespread use during the time of analysis, the decrease of cancer deaths was attributed mainly to the introduction of PKIs.

The first-generation inhibitors, gefitinib and erlotinib, for treating non-small cell lung cancer (NSCLC) target the epidermal growth factor receptor (EGFR). Patients benefiting from these drugs harbor pathogenic mutations. Approximately 15% of patients suffering from NSCLC carry oncogenic mutations in EGFR.^6,7^ Two common genetic alternations are exon 19 deletions and the point mutation L858R in the kinase domain.^6–8^ However, many other mutations have been found from genetic/genomic testing of patients suffering from lung cancer. For example, our analysis of the data for lung cancer patients (multiple subtypes) in cBioPortal^9–11^ and COSMIC^12,13^ found close to 800 unique mutations in the kinase domain. Many of these mutants have not yet been classified or classified as Variant of Uncertain Significance (VUS) and are therefore not actionable. It is also possible that new mutants will continue to be found. Predictive computational methods can help prioritize mutants to be studied by experimental/clinical means to find those that are disease-driving and can be treated by existing drugs or those that can become targets for future drug development. Currently, computational predictions are only considered as supporting evidence for somatic mutations according to the Standard Operating Procedure (SOP) of the Clinical Genome Resource/Cancer Genomics Consortium/Variant Interpretation for Cancer Consortium (ClinGen/CGC/VICC).^14^ However, for germline mutations, ClinGen^15^ considers some newer computational tools as providing moderate or strong evidence.^16^ These tools include REVEL,^17^ BayesDel,^18^ MutPred2,^19^ and VEST4.^20^ It will be fruitful to find computational tools that can be elevated to providing stronger evidence for somatic mutations as well. Existing methods use features such as evolutionary conservation, frequency of occurrence in the general population, cancer mutational hotspots, and structural properties of mutated sites (such as solvent accessibility, structure and function of neighborhood, and proximity to protein–protein interface). On the other hand, these methods usually do not explicitly account for changes in the conformational dynamics of the protein induced by mutations. Models that include these features explicitly could add a useful piece of complementary information to improve prediction and to help rationalize how a mutation could induce a conformational shift to trigger oncogenicity. In this work, we tested the hypothesis that structural drifts observed from molecular dynamics simulations could help to distinguish oncogenic from benign mutants with the help of machine learning. Here, we worked toward a modest goal of identifying an algorithm applicable to a specific protein (EGFR) and a specific disease (non-small cell lung cancer). This should already be useful as many patients are affected by this disease for which therapeutic intervention could be feasible by targeting oncogenic mutants of this protein with drugs with fewer side effects and lower toxicity than chemotherapy.

To test our hypothesis effectively, we included diverse mutants that have been observed and that have useful information supporting their classification as oncogenic or benign. While the classical mutations L858R and exon 19 deletions are well studied and known to cause disease, some mutations are rare and do not have as much information supporting their classification. Rare mutations are more difficult to detect. For example, the traditional polymer chain reaction (PCR) often misses these mutants. Fang et al.^21^ and Chang et al.^22^ found that next-generation sequencing was more effective in finding these rare mutants from patient samples, but these tests are not yet commonly ordered for patients. Therefore, many rare mutations have been observed only a few times or even just once, and less is known about the oncogenicity of these mutants. Nevertheless, we have included several rare mutations in this initial proof-of-concept study (Table 1). As more data become available, our study can be repeated and reevaluated.

**Table 1:** Wild-Type and Mutants of EGFR Simulated in This Study

Protein	Source	Label in machine learning
wild-type		Benign
R831H	OncoKB^29,30^	Benign
F712L	OncoKB^29,30^	Benign
I744M	OncoKB^29,30^	Benign
S720F	OncoKB^29,30^	Benign
V774L	OncoKB^29,30^	Benign
P741S	OncoKB^29,30^	Benign
I926N	ClinVar SCV001661111 ^31^	Benign
I966V	ClinVar RCV000987887.1 ^31^	Benign
V904I	ClinVar SCV001649376 ^31^	Benign
Δ747,Δ752,P753S	BindingDB^32^	Oncogenic
Δ747-E752,P753S	BindingDB^32^	Oncogenic
Ins769-SV-770	Tamirat et al.,^33^ Yan et al.^34^	Oncogenic
Ins769-SVD-770	Yang et al.,^34^ Zhao et al.^35^	Oncogenic
Ins770-NPG-771	Yasuda et al.,^27^ Zhao et al.^35^	Oncogenic
ΔE746-A750	CIViC^36^	Oncogenic
ΔE746-A750,T790M	OncoKB^29,30^	Oncogenic
G719A,L861Q	Robichaux et al.^28^	Oncogenic
G719C	CIViC^36^	Oncogenic
G719S	CIViC^36^	Oncogenic
G724S	Robichaux et al.,^28^ Wang et al.^37^	Oncogenic
G724S,T790M	Robichaux et al.,^28^ Wang et al.^37^	Oncogenic
ΔL747_E749,A750P	Zhao et al.,^35^ Truini et al.,^38^ Di Federico et al.^39^	Oncogenic
L747-T751>P	CIViC^36^	Oncogenic
L858R	CIViC^36^	Oncogenic
L858R,L718V	Robichaux et al.^28^	Oncogenic
T790M,L858R,C797S	Robichaux et al.^28^	Oncogenic
T790M,V843I,L858R	Robichaux et al.^28^	Oncogenic
L861Q	Robichaux et al.^28^	Oncogenic
S768I,V769L	Niogret et al.^40^	Oncogenic
T751_I759>N	Chang et al.,^22^ Schrock et al.,^41^ COSMIC COSV51804832 ^13^	Oncogenic
T790M	CIViC^36^	Oncogenic
T790M,L858R	CIViC^36^	Oncogenic

Figure 1a shows the locations of the point mutations, and Figure 1b shows the locations near the insertion/deletion sites for the proteins studied in this work. These mutations were found in several parts of the kinase domain, including the phosphate-binding loop (P-loop), C-helix, activation loop (A-loop), and DFG motif. The structural rationale for oncogenicity or benignancy is clearer for some than the others. The well-known L858R found in the A-loop was believed to destabilize the inactive form by opening the A-loop for binding substrates. A molecular dynamics study suggested that the ubiquitous exon 19 deletion removed part of the β3−αC loop, rigidifying and stabilizing the C-helix in the active conformation with the K745–E762 catalytic salt bridge formed.^23^ However, recent studies found that different exon 19 deletions could affect the kinase domain in different ways, leading to different degrees of oncogenicity, with different drug responses.^24^ Dimerization is utilized by the wild-type protein to mediate cell signaling. However, different mutants depend on dimerization in different ways. For example, the L858R mutant is dependent on dimerization to activate EGFR.^25^ The double mutation L858R/T790M, many exon 19 deletions, and exon 20 insertions are not dimerization-dependent.^26^ Different mechanisms are used by exon 20 insertions to activate EGFR. Some exon 20 insertions introduce bulkiness right after the C-helix. Bulkiness acts like a wedge preventing the C-helix from rotating from active to inactive conformations.^27^ The A763_Y764insFQEA mutant was found to shift the register of the C-helix in the N-terminal direction.^27^ Different exon 20 insertions were found to have diverse responses to drugs.^28^ These examples illustrate the heterogeneity and complexity on how different mutants of EGFR alter structures in different ways to present oncogenicity; many fine details remain to be discovered. Although the motivation of this study was to develop a machine-learning model that could learn from existing mutants and predict the oncogenicity of new mutants even before the detailed molecular mechanisms were known, this research provided some useful insights into molecular mechanisms that simulations lasting 3 μs could provide.

**Figure 1:**
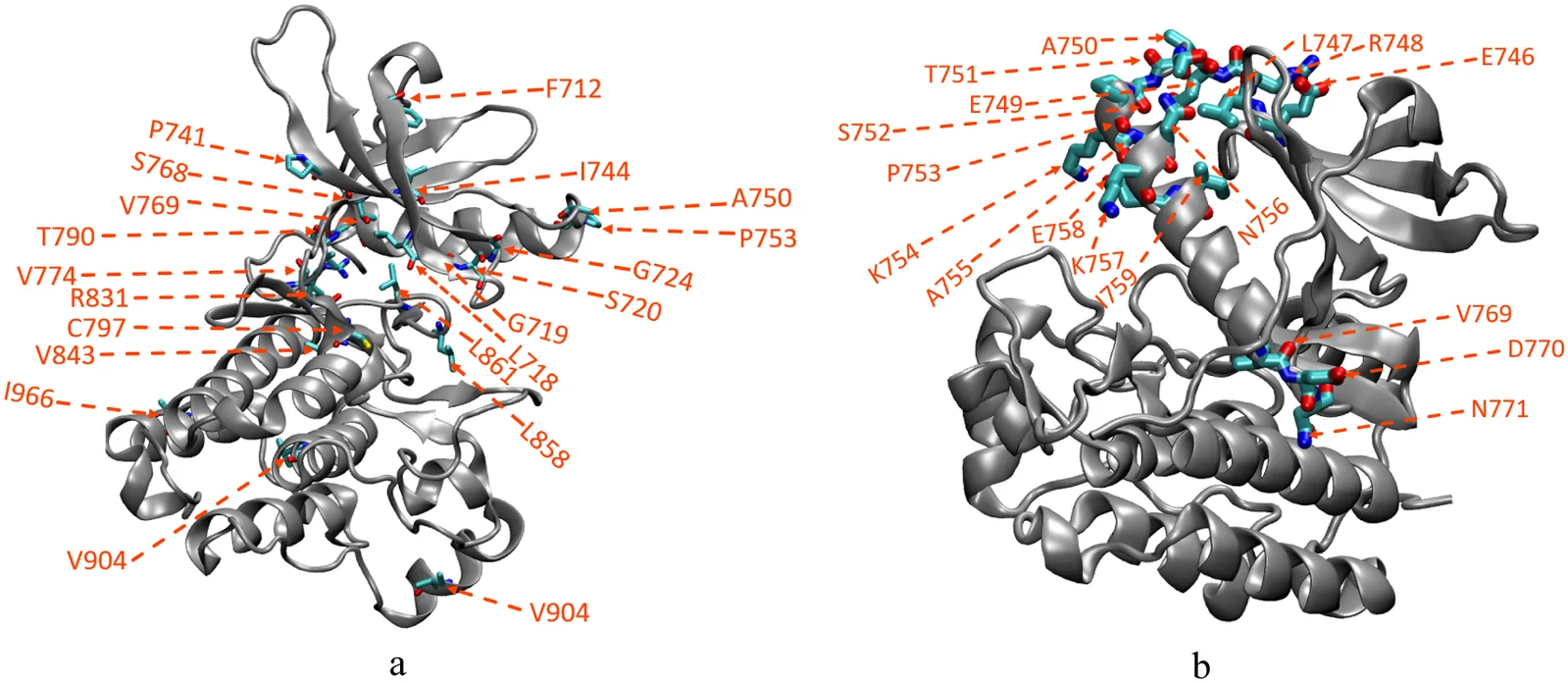
Location of alternation sites of EGFR mutants included in this study. a) Location of point mutations. b) Location near deletion/insertion sites.

## Methods

### Molecular Dynamics Simulation (MDS)

Molecular dynamics simulations were conducted on wild-type EGFR and thirty-two mutants. The mutants included nine benign and twenty-three disease-driving variants, as shown in Table 1. All of the simulations started from the active structure in PDB entry 3VJO.^42^ We started from the active form to evaluate whether a mutation could cause EGFR to be trapped in the active form and therefore make the protein constitutively active even without an external cue. Crystallographic water molecules, the bound ATP analog AMPPNP, and the Mg^2+^ ion were removed. The MODELLER package^43–45^ was used to make the mutations and fill in the missing residues in the crystal structure. Every mutant started with the same crystal structure. For point mutations, all residues were kept fixed, except for the residues that were replaced. For deletion, insertion, or adding missing short loops, two residues flanking deletion, insertion, or a short missing loop on each side were allowed to move so that MODELLER could generate strain-free structures.

The CHARMM36m force field^46–48^ was employed to simulate each system. After adding hydrogens by CHARMM,^49^ each system was minimized under harmonic restraints on the non-hydrogen atoms of the protein to the initial structure using the GBMV implicit solvent model.^50,51^ Each system was then solvated in a rhombic dodecahedral box with the TIP3P water model.^52^ Molecular dynamics simulations of all systems were conducted using NAMD.^53,54^ A switching function for nonbonded interactions between 10 and 12 Å and the particle mesh Ewald method were used (Essmann et al., 1995). Afterward, each system was minimized again using harmonic restraints applied to non-hydrogen atoms of the protein with the initial structure as the reference; a force constant of 10 kcal/mol/Å^2^ was used. We then gradually heated the system from 10 to 300 K over 1.5 ns, with the temperature increased by 10 K every 50 ps. SHAKE^55^ was applied to all bonds containing hydrogens to allow the use of a larger time step of 2 fs. A Langevin bath was used for temperature control. The system was then equilibrated at constant pressure over 1 ns in five phases of 200 ps each, using harmonic restraints of 5, 1, 0.5, 0.1, and 0 kcal/mol/Å^2^. The system was then run for three replicate simulations of 1 μs each, starting from distinct initial velocities to obtain independent simulations.

We first examined some structural features from the molecular dynamics simulations individually to gain insights into whether obvious cutoff values could be used to separate the proteins into oncogenic or benign ones. If obvious cutoff values cannot be used, then we examined whether machine learning could help to use these features more intelligently to separate the proteins. We then examined whether the use of combinations of these features could improve the separation of the proteins.

One feature monitored was Root-Mean-Square Deviation (RMSD) from the initial crystal structure representing an active form of the protein. The motivation was that oncogenic mutants might be trapped close to the active form and have low RMSD, while benign mutants might drift further away from the active form and therefore give larger RMSD for the same simulation length. The initial structure derived from PDB entry 3VJO^42^ was induced to be in the active form by the binding of the ATP analog AMPPNP. Here, we assumed that the conformational space of the active form to be much smaller than that of the inactive form because specific conformations were necessary for function, and the structure in 3VJO represented this active conformational space reasonably well. This assumption stemmed from the crystallographic studies by the Kuriyan group.^56^ Besides examining RMSD averaged over the last 100 ns of each simulation, we also computed the fraction of time a protein had RMSD below 2 Å. The latter reflected the population of structures in the simulations that resembled the active structure and could be a better proxy on whether a protein was oncogenic than by simply averaging a wide range of structures from molecular dynamics simulations. For this purpose, one wanted to choose a cutoff value such that the fraction covered a good dynamic range for comparing the results from the different proteins. We started off with a cutoff of 1.5 Å and obtained a narrow dynamic range of 0 to 0.1 out of the full range between 0 and 1. This was not surprising because molecular dynamics simulations often drifted away from the starting crystal structures partly because simulations were not carried out in the crystal environment and partly because of approximations such as force fields used in molecular dynamics simulations. However, when we increased the cutoff to 2.0 Å, we obtained a good dynamic range from 0.1 to 0.8 among the proteins. This covered almost the whole range from 0 to 1 and was adequate for this study.

As the Root-Mean-Square Fluctuation (RMSF) of the residues of a protein is sensitive to the structure of the protein, we also examined whether it could be used to distinguish oncogenic from benign proteins. The radius of gyration was also monitored to examine whether it differed between the two classes of proteins. To probe collective motion, we performed principal component analysis and projected the trajectories onto the first two principal components to examine whether the two classes of proteins presented significant differences in sampling this low-dimensional space. We performed the principal component analysis in distance space with the covariance matrices constructed from the distances between α carbons. Only distances observed within 10 Å in at least one frame of the simulations were included. To compare the conformational densities between two proteins, we divided the space spanned by the first two principal components into a 1.5 Å grid. Using the conformational densities projected onto the grids, we computed the similarity between every pair of proteins. Only grids containing more than 3% of the frames in at least one simulation of the proteins were included in computing the similarity. We calculated similarities in two ways: (1) by root-mean-square deviation; (2) by the Jensen–Shannon divergence^57^ of the projected densities. The similarities were used to cluster the proteins to examine whether benign and oncogenic mutants segregated into different clusters.

To provide finer probes on local structural change, we computed the fraction of time each residue spent in the α helical or the β state, as determined by DSSP.^58^ In the machine-learning models, we also used intramolecular hydrogen bonds and residue contacts as features. We calculated the fraction of time each intramolecular hydrogen bond was formed. The Baker–Hubbard function in MDTraj^59^ was used to search for hydrogen bonds based on a distance criterion of 2.5 Å between hydrogen and acceptor atoms and an angular criterion of no more than 60° away from straightness. Only hydrogen bonds involving the side chain of at least one residue were considered. Residue contacts were defined using a cutoff of 4 Å between heavy atoms. For both hydrogen bonds and residue contacts, we only considered those that were present between 25 and 75% in at least one system, to focus on those more likely to distinguish benign from oncogenic proteins.

As Modi and Dunbrack^60,61^ had clustered experimental structures of the kinase domain and developed a set of structural features to distinguish active from inactive conformations, we applied these features to our trajectories to calculate the population of structures within a simulation that adopted the active form. The set of features include the ϕ and ψ angles of the aspartate and the phenylalanine in the DFG motif as well as the residue, a threonine, to its N-terminal end, and the χ_1_ angle of the phenylalanine.

### Machine Learning Using Structural Features Obtained from Molecular Dynamics Simulations

For machine learning, we used the random forest model (RFM)^62^ in the scikit-learn package.^63^ The RFM uses an ensemble of decision trees. To avoid overfitting, we set the maximum depth of each tree to 3. We determined the optimal number of trees by minimizing the out-of-bag error. In addition, the class_weight=’balanced’ setting in scikit-learn was used to increase the weight on benign mutants compared to oncogenic ones as we had fewer benign than oncogenic mutants. The performance of the model was measured by the Area under Receiver Operating Characteristic Curve (AUC).

We also performed cross-validation to check whether the machine-learning models were not overfitted. In addition, we used bootstrapping to estimate the uncertainties resulting from finite sampling in the molecular dynamics simulations. The error bar of the AUC for each model thus reflected both the uncertainty resulting from variation in choosing training and testing proteins as well as sampling fluctuations from finite-molecular-dynamics simulations. To this end, we performed ten rounds of 3-fold cross-validation using bootstrapping trajectories from the molecular dynamics simulations. This was done in a stratified way such that each training or test set contained trajectories from benign or oncogenic mutants in the same proportion as the data set as a whole. The StratifiedGroupKFold cross-validation iterator object in scikit-learn was used. The mean and standard error of the AUC were then calculated. The Nadeau–Bengio correction^64^ was used in calculating the standard error to account for correlation of models randomly generated from the data during cross-validation so that statistical errors were less likely to be underestimated. We also note that by default scikit-learn uses bootstrapping to construct subsamples of the data to build the individual decision trees that make up the random forest model; this bootstrapping is distinct from the bootstrapping of molecular dynamics simulations, which was implemented outside of scikit-learn. The bootstrapping of the molecular dynamics simulations was done by randomly selecting three trajectories for each protein with replacement. In identifying the most important subset of features in the RFM, we found the features that reduced the Gini impurity of the trees the most.

### Monitoring Common Structural Features of the Kinase Domain

As scientists often examine several common structural features of the kinase domain,^65–68^ we also monitored them to help interpret results from the machine-learning models. One is the C-helix. The *in* conformation is believed to be occupied by active proteins, whereas the *out* conformation is thought to be occupied by inactive proteins. The salt bridge between K745 and E762 is formed in the *in*-conformation and was monitored in our simulations.

The DFG motif is another common structural motif analyzed. The DFG-in conformation is believed to be adopted by active proteins and the DFG-out conformation by inactive proteins. We made Ramachandran plots for residues 854–857 (including T854, which is immediately before the DFG motif) covering this motif as a function of time. We included T854 because Modi–Dubrack^60^ found this to be useful for distinguishing between active and inactive conformations. Likewise, we included the χ_1_ angle of the phenylalanine as it is also used in the Modi–Dubrack model.

As dimerization is a mechanism for activation in the wild-type protein, we also monitored the surface exposure of the residues at the interface during the simulation of the monomers. As there was only one kinase domain in the starting structure for our simulations, we used a different structure (PDB code 3GOP),^69^ which shows two kinase domains oriented in the dimerized conformation, to identify the dimer interface. The residues making up the dimer interface were identified from this structure by selecting all residues in each domain with at least one heavy atom within 5 Å of the other domain. The solvent-accessible surface area (SASA) of the residues in the N-terminal and C-terminal portions of the interface was computed with the Shrake–Rupley method in MDTraj.^59^

## Results and Discussion

We organized this section first by analyzing some features to examine whether they could obviously be used to distinguish between oncogenic and benign proteins. We then examined whether machine learning could use the features more intelligently to distinguish between the proteins. Finally, to gain insights into why the machine-learning models selected or did not select features associated with several common structural motifs for classification, we analyzed these motifs from the molecular dynamics simulations.

### Can Individual Structural Features with Simple Cutoff Values Distinguish between Oncogenic and Benign Proteins?

Figure S1 shows the backbone Root-Mean-Square Deviation (RMSD) as a function of time for each of the three 1-μs simulations for each protein simulated. Table S1 reports the RMSD averaged over the last 100 ns of the three trajectories of each protein with the error bars shown. It does not appear that using a simple cutoff value of this averaged RMSD could distinguish between the two classes of proteins well. If oncogenicity can be encoded by this feature in a more complex manner, it needs a more sophisticated method to sort out. As will be shown later, machine learning using RMSD in another manner was among the best single features that gave good predictive performance.

Another point concerns sampling statistics. The standard error of the average RMSD shown in Table S1 gave a range between 0.06 Å and 0.5 Å. Although some proteins showed relatively similar RMSDs from their three trajectories with standard errors as low as 0.06 Å, some displayed larger differences, up to 0.5 Å. However, the goal of the current study was not to compute precise ensemble-averaged properties but to examine whether features obtained from molecular dynamics simulations with sufficient length could be used to distinguish between oncogenic and benign proteins. An analogy is to ask *n* blind people to touch an animal and use their collective information to tell whether it is an elephant or not, after giving a training set of animals. They might not be able to tell the precise dimension of the animal, but they might be able to tell whether the animal is an elephant or not if *n* is large enough. As shall be seen below, using suitable features with the aid of machine learning could help to distinguish oncogenic from benign proteins using our molecular dynamics simulations totaling 3 μs for each protein.

Table S1 also shows results obtained by calculating the fraction of time a protein stayed within 2 Å of the initial active structure. This factor might reflect enzymatic activity better than the average RMSD does because it better represents the fraction of time the protein remains close enough to the active conformation to be enzymatically active. As will be seen below, machine learning using this feature alone could distinguish the two classes of proteins better than several other features used individually.

Table S1 also reports the average radius of gyration of the proteins computed from the trajectories. It covered a narrow range between 19.74 ± 0.04 and 20.00 ± 0.06 Å. This suggested that all the proteins maintained a relatively similar compact structure, and this feature might not be useful to distinguish the two classes of proteins. The time course of the radius of gyration for all proteins is shown in Figure S2.

Table S1 also shows the mean α helical (ranging from 17.5 ± 0.3% to 19.3 ± 0.2%) and β content (ranging from 35.2 ± 0.2% to 36.9 ± 0.2%) of the proteins. The time dependence is shown in Figure S3. These numbers did not vary substantially from protein to protein and might not be useful for distinguishing the two classes of proteins. However, averaging over all the residues could lose a lot of information. In machine learning, we used the secondary structural content at the residue level and found it to be a useful feature for separating oncogenic from benign proteins (see below).

Figure 2 shows the fraction of time each protein spent in the active form using the criteria defined by Modi and Dubrack.^60^ The results were ordered by numerical values, and the benign proteins are displayed in bold on the *y*-axis. The results suggested that this quantity did not distinguish the oncogenic proteins from the benign proteins well. As shown below, this was still true, even with the aid of machine learning.

**Figure 2:**
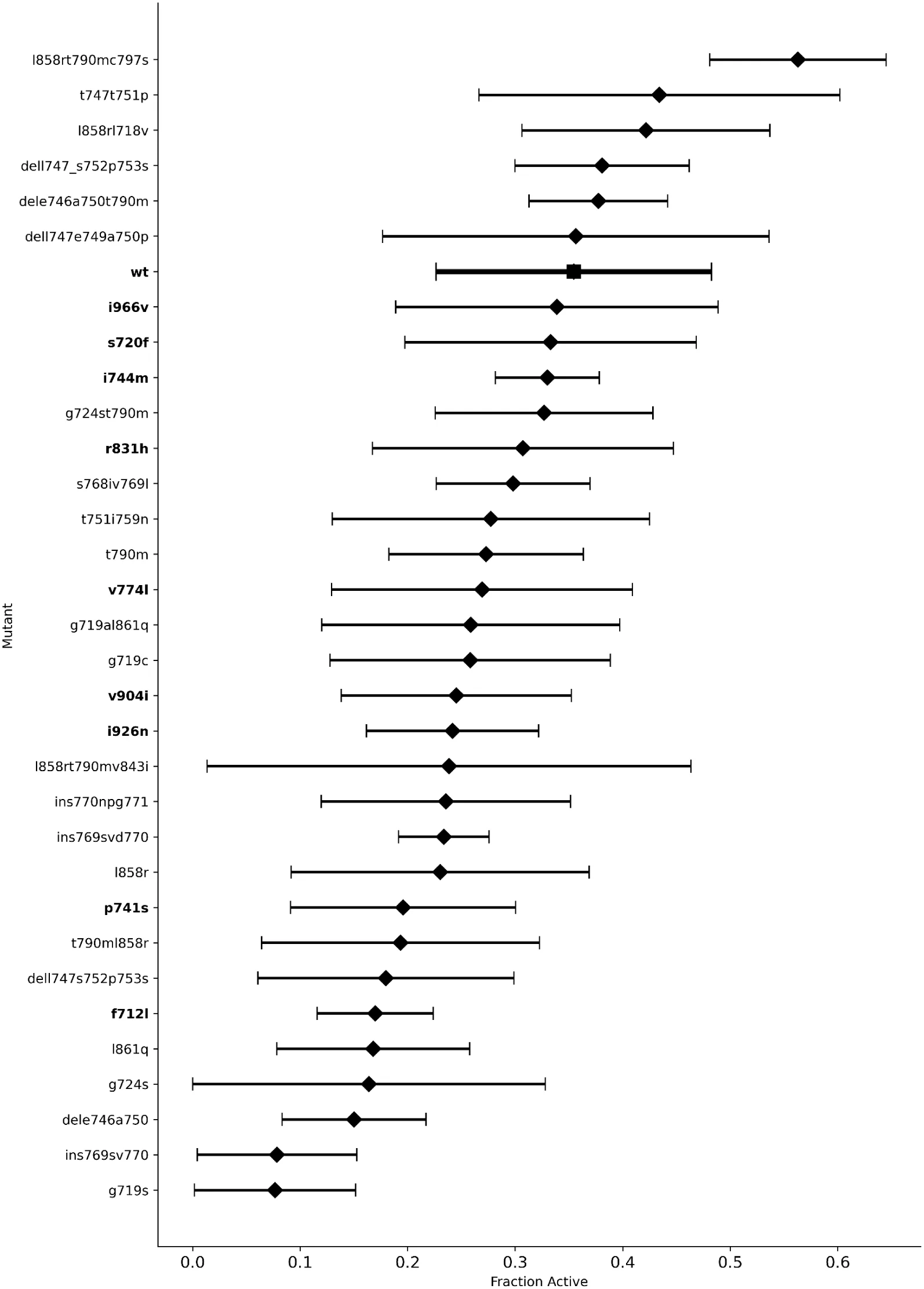
Fraction of time a protein spent in the active form, using the criteria of Modi and Dunbrack.^60^ Error bars were standard errors obtained from the three 1 μs trajectories of each protein.

Figure S4 shows the root-mean-square fluctuations (RMSFs) of the proteins. This feature did not vary significantly among the proteins. We therefore did not use this feature in the machine-learning models.

Figure 3 displays the projection of the trajectories of each protein onto the first two principal components (a. for benign proteins and b. for oncogenic). The figure did not reveal a significant difference in the space covered. Collectively, the oncogenic proteins sampled a larger space, but this might result from the larger number of oncogenic proteins simulated. To better compare the conformational sampling between proteins, we divided the space spanned by the first two principal components into grids, as described in Methods. We then computed the similarities in the sampled distribution between pairs of proteins using the population densities of these grids. The similarities were used to perform hierarchical clustering to examine whether the oncogenic and benign proteins clustered separately. Figure 4 presents results in which the similarity between two proteins were obtained by the root-mean-square differences between their population densities in the grids. The benign proteins were intermixed with the pathogenic proteins, making it difficult to use this feature alone to separate the two classes of proteins. We also used the Jensen–Shannon divergence as the similarity measure (Figure S5) but obtained a similar qualitative conclusion.

**Figure 3:**
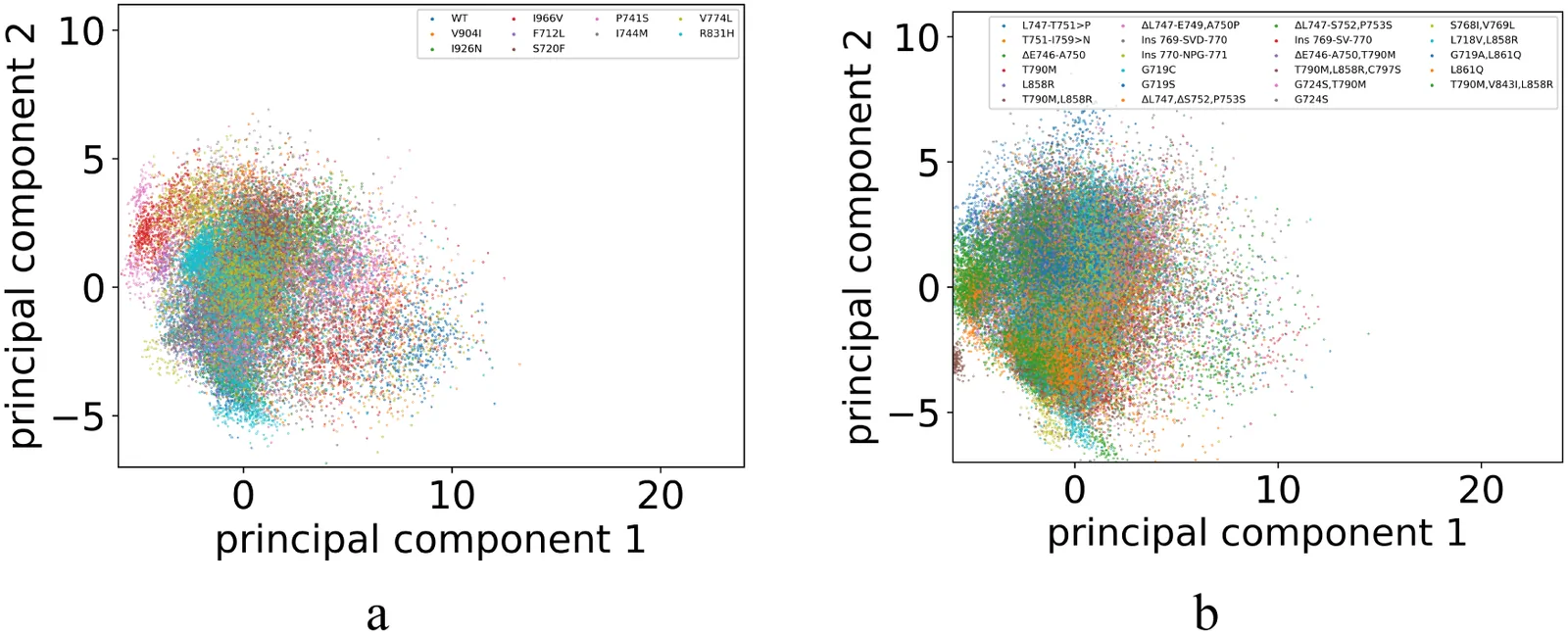
Projection of dynamic snapshots on the first two principal components. (a) Benign proteins and (b) oncogenic proteins.

**Figure 4:**
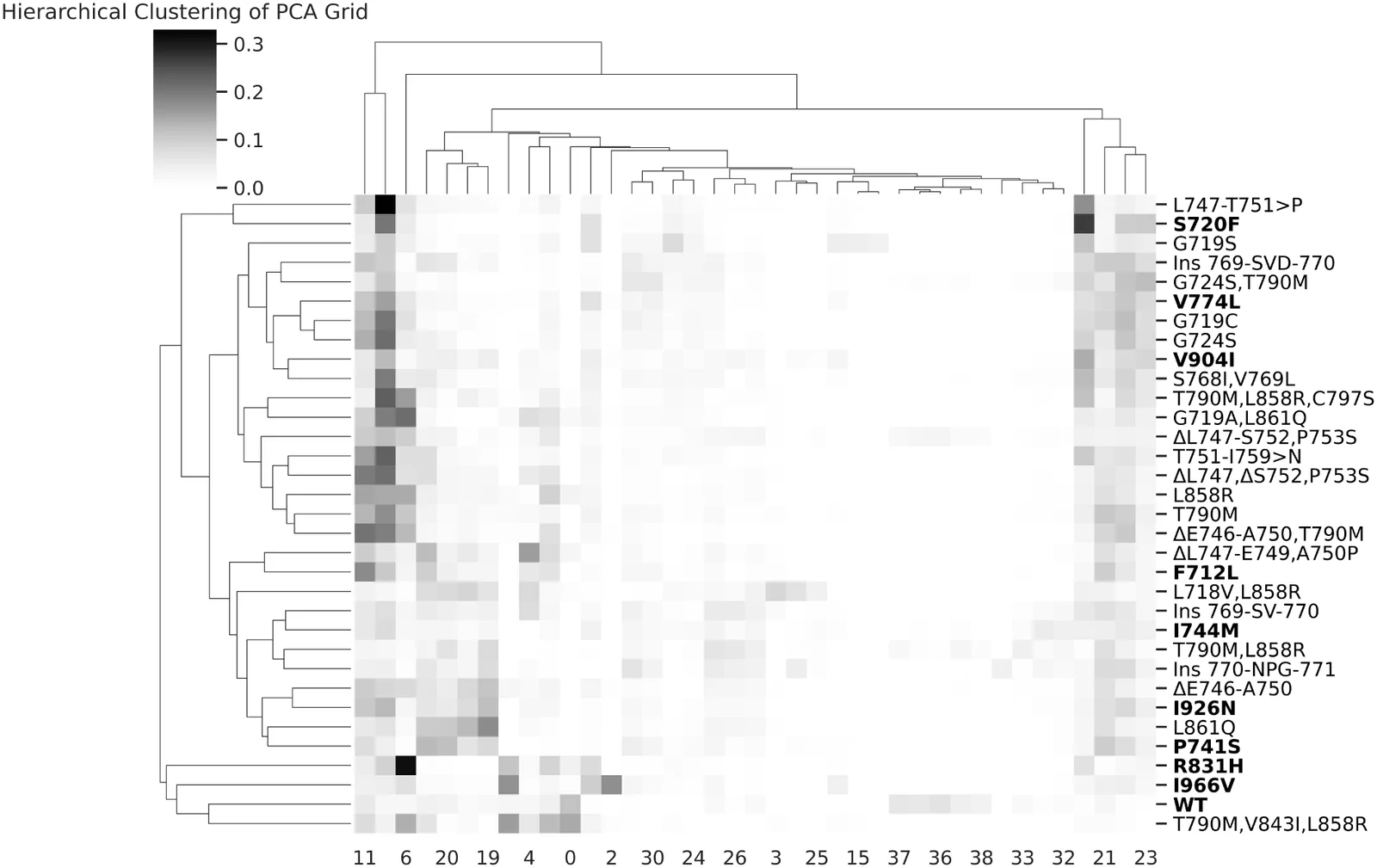
Hierarchical clustering of the proteins based on the conformational densities projected onto the first two principal components. The clustering was performed by using as a similarity measure the root-mean-square deviation between the conformational densities of two proteins in grids formed by the first two principal components. (Labels of benign mutants are shown in bold.)

### Machine-Learning Models Using Features Obtained from Molecular Dynamics Simulations Could Substantially Improve the Distinction between Oncogenic and Benign Proteins

There are various reasons why using simple cutoffs on individual features obtained from molecular dynamics simulations may not be adequate for classifying EGFR mutations as benign or oncogenic. The distribution of any given dynamical feature may differ between benign and oncogenic mutants in a complex way that may go beyond what can be represented with a single cutoff. Additionally, the correlated distributions of features may also differ between benign and oncogenic mutants in ways that cannot be adequately represented with combinations of single cutoff values. The decision trees that make up the random forest models used here allow for the possibility of classifying mutants based on multiple features of the dynamics using multiple cutoffs for each feature. We began by considering models that used only one feature or type of feature and then tested models that used two or more types of features.

As discussed in the Methods section, the performance of each model was measured by the area under receiver characteristic curve (AUC). The confidence interval was estimated from the standard error with the Nadeau–Bengio correction.^64^ The results are summarized in Table 2.

**Table 2:** Summary of the Performance of Machine-Learning Models Using Different Features^a^

Features	Mean AUC
Fraction of each residue in α or β secondary structure	0.67 ± 0.07
Fraction of snapshots in active form	0.6 ± 0.1
Fraction of snapshots within 2 Å of initial active conformation	0.8 ± 0.1
Fraction of each hydrogen bond formed during the trajectories	0.59 ± 0.09
Fraction of each residue contact formed during the trajectories	0.8 ± 0.1
Conformational densities projected onto the first two principal components	0.6 ± 0.1
Fraction of snapshots in active form and best subset of secondary structures^#^	0.8± 0.1
Fraction of snapshots in active form and fraction of each residue in α or β secondary structure	0.8 ± 0.1
Fraction of snapshots in active form and fraction of snapshots within 2 Å of initial active conformation	0.75 ± 0.07
Fraction of snapshots in active form, fraction of snapshots within 2 Å of initial active conformation, and fraction of each residue in α or β secondary structure	0.8 ± 0.1
Fraction of snapshots in active form and fraction of time each residue contact is formed	0.7 ± 0.1
Fraction of snapshots in active form and fraction of time each hydrogen bond is formed	0.64 ± 0.09
Fraction of snapshots within 2 Å of initial active conformation and fraction of each residue contact formed during the trajectories	0.51 ± 0.08

a # means only the most important subset of residues identified by the random forest model was included when using as features the residue-level α and β secondary structural occupation from the molecular dynamics simulation of the proteins.

The two types of features that gave the best predictions when considered individually were the fraction of time a protein stayed within 2 Å of the starting active structure and the fraction of time individual residue contacts were formed. The AUCs reached 0.8. We also tried several combinations of features but did not find an AUC better than 0.8. Although using the fraction of snapshots within 2 Å as a feature or the residue-contact features individually gave good AUC values of 0.8, combining them gave a lower AUC of ∼0.5. This likely resulted from feature dilution. The two features probed different mechanisms. Using each feature alone gave good results because it could utilize each mechanism to a fuller extent. Mixing the two can dilute the impact of each feature. For example, if the first split in a tree uses the first feature, it could already divide the samples into classes not favorable to be handled by the second feature in the next split. Nevertheless, the model combining residue-level secondary structures and fraction in the active form gave among the best AUC of 0.8 ± 0.1. Because this model included features at the residue level, we took advantage of it to help identify the most useful regions of the proteins for distinguishing oncogenic from benign proteins. The receiver operating characteristic curves of the models using these two features are shown in Figure 5. The receiver operating characteristic curves for the other models are shown in Figures S6–S8.

**Figure 5:**
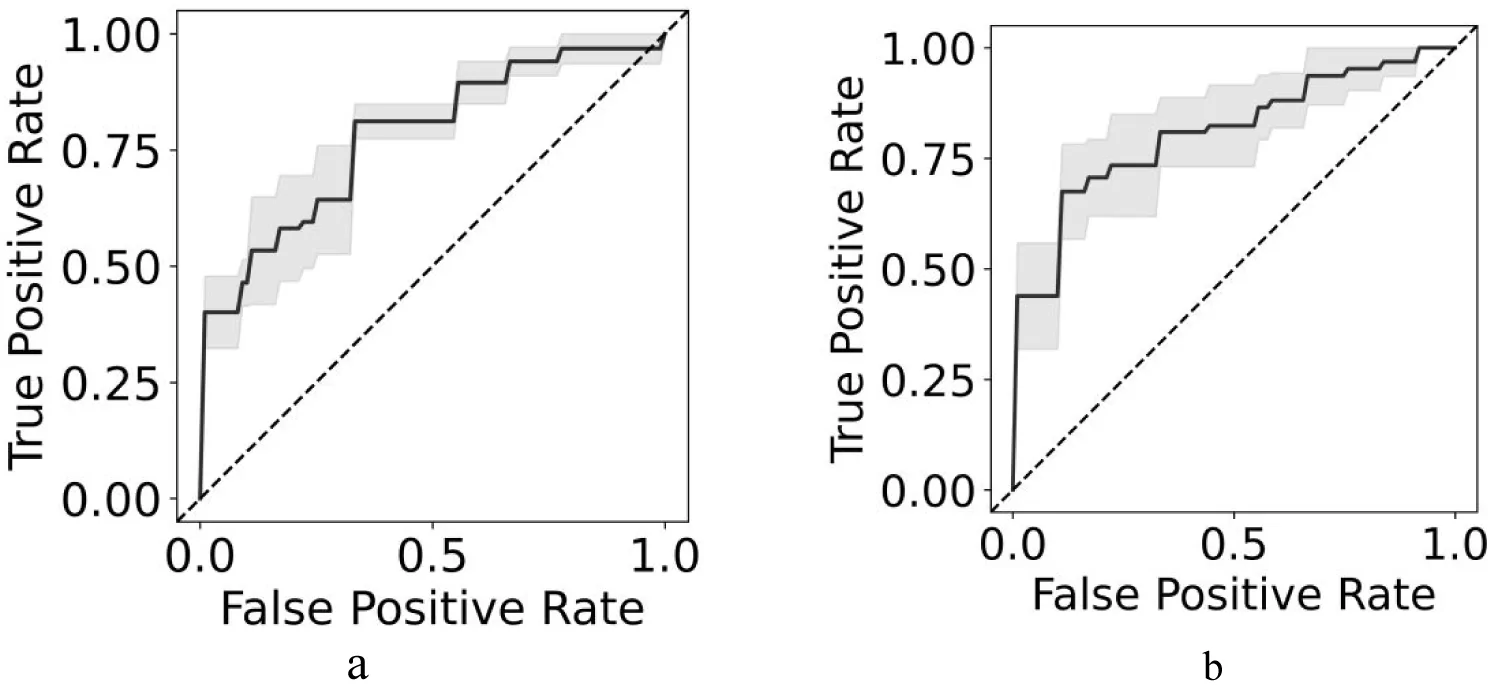
Receiver operating characteristic curves of the machine-learning models using (a) the fraction of time in the active form and the fraction of time each residue in α helical or β structure, (b) as in (a), but using only the most important residues identified by the random forest model when calculating the residue-level secondary structural content.

As one of the best models used the secondary structural content at the residue level, it provided a way to identify the parts of the proteins whose features were most useful for distinguishing between oncogenic and benign proteins. We achieved this by identifying the features in the model that decreased the Gini impurity the most in the random forest model, as described in the Methods. The top 10 residues are displayed in Figure 6a, along with the structure of the kinase domain.

**Figure 6:**
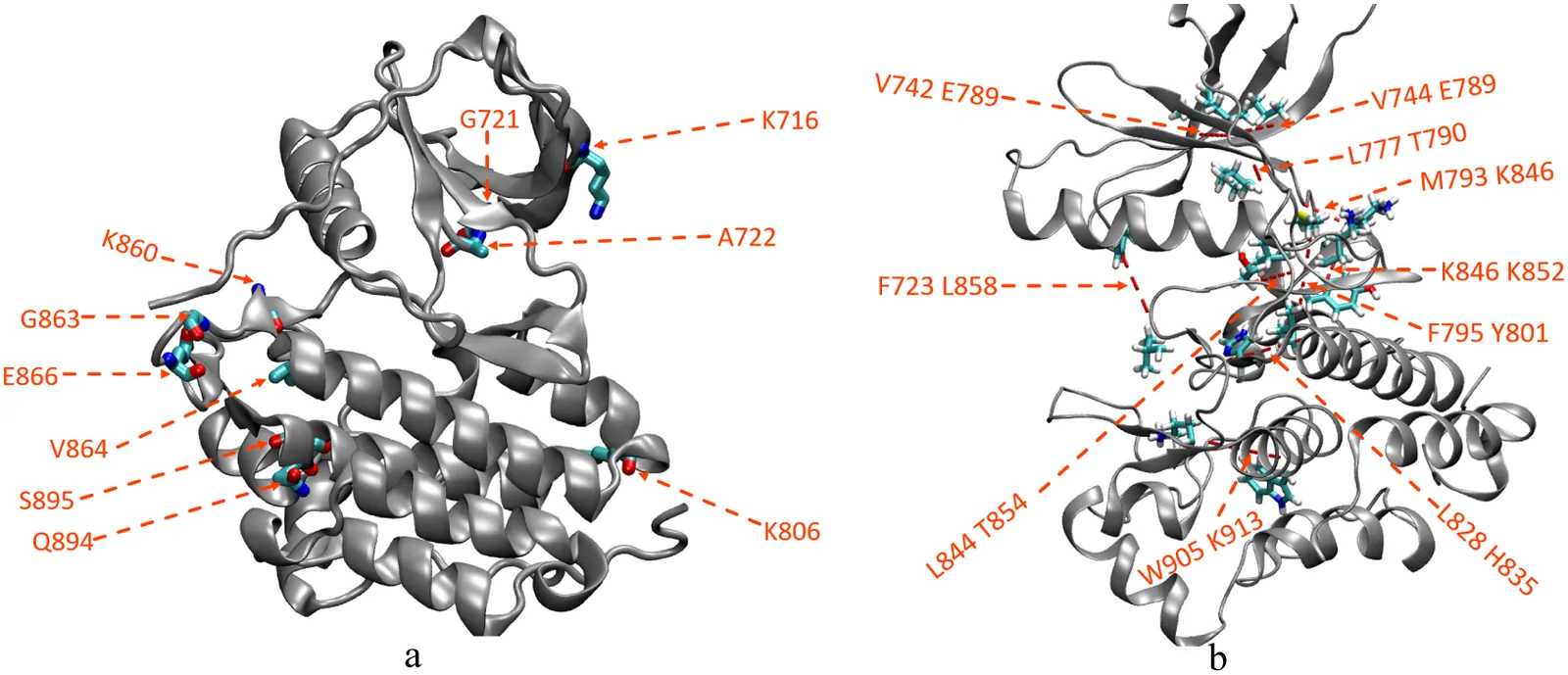
(a) Top ten residues identified to be most useful for distinguishing the oncogenic proteins from the benign ones using the random forest model. b) Top ten most useful residue contacts identified from the random forest model.

G721 and A722 are in the glycine-rich loop important for binding ATP. K860, G863, V864, and E866 are part of the activation loop just after the DFG motif (residues 855–857). The activation loop is of course important in function. It is interesting that although the machine-learning models were not specifically supplied with information on common structural motifs in protein kinases, they identified these parts of the proteins as useful in distinguishing between oncogenic and benign proteins. On the other hand, the machine-learning models did not select the salt bridge reflecting the *in*/*out* conformation of the C-helix, nor parameters associated with the DFG motif. This does not mean that these features are not useful or important in general; it might just mean that simulations lasting 3 μs were not long enough to observe the conformational switch between the *in* and *out* conformations of the C-helix and of the *in* and *out* conformations of the DFG motif. Accordingly, the machine-learning models relied on other useful features to draw conclusions; a model just needs to use enough features. This was learned from developing the Modi–Dunbrack model as well: the model only needs to use several structural parameters in or near the DFG motif to classify proteins into active or inactive conformations; it does not need to use information from other motifs.

As the random forest model using residue contacts also gave a good AUC, we used it to identify the most useful residue contacts in classifying the proteins (Figure 6b). Many of these involved residues were found in exon 19 where deletions occurred and/or exon 20 where insertions occurred (V742-E789, V744-E789, L777-T790, M793-K846, V795-Y801). Because deletions or insertions introduce rather large structural changes, residue contacts in this region could be useful for distinguishing oncogenic from benign proteins. It is also not surprising that the random forest model takes advantage of other residue contacts. F723-L858 could monitor the conformational change of the activation loop. L844-T854 and K846-K852 occurred at the N-terminal end of the DFG motif. The Modi–Dunbrack model already used T854 to classify active and inactive forms of protein kinases. K852 nearby could be useful too, not only by simply monitoring Ramachandran maps but also by monitoring the movement of the DFG motif relative to the neighboring part of the protein. L828-H835 involved the histidine in the catalytic HRD motif, and the random forest model could use this residue contact to monitor the active and inactive states of the mutants.

### Analyses of Functional Motifs from Molecular Dynamics Simulations

To gain further insights into why the machine-learning models selected or did not select features associated with several common structural motifs for classification, we analyzed these motifs from the molecular dynamics simulations.

#### Salt Bridge and C-Helix In/Out Conformation

Table 3 shows the fraction of time the salt bridge was formed in different proteins This salt bridge is formed when the C-helix adopts the *in*-active conformation. From the data, it was not obvious that this quantity could help distinguish between the two classes of proteins. The oncogenic proteins did not consistently present a higher probability of forming this salt bridge than the benign proteins. This might explain why the machine-learning model did not pick this as a top feature. Notably, the Modi–Dunbrack model did not utilize this feature to distinguish active and inactive conformations. It mainly uses features in or around the DFG motif.

**Table 3:** Fractional Occupancy of Salt Bridge Formed between K745 and E762

Benign	Occupancy	Standard Error	Oncogenic	Occupancy	Standard Error
Wildtype	0.92	0.02	L747-T751>P	0.89	0.04
V904I	0.86	0.09	T751-I759>N	0.6	0.3
I926N	0.7	0.2	ΔE746-A750	0.4	0.2
I966V	0.7	0.2	T790M	0.94	0.01
F712L	0.7	0.3	L858R	0.6	0.3
S720F	0.8	0.1	T790M,L858R	0.6	0.3
P741S	0.5	0.3	ΔL747-E749,A750P	0.6	0.3
I744M	0.7	0.1	Ins769SVD770	0.6	0.1
V774L	0.6	0.3	Ins770NPG771	0.6	0.3
R831H	0.8	0.1	G719C	0.6	0.3
			G719S	0.3	0.3
			ΔL747ΔS752,P753S	0.5	0.2
			ΔL747-S752,P753S	0.96	0.02
			Ins769-SV-770	0.3	0.3
			ΔE746-A750, T790M	0.982	0.006
			T790M,L858R,C797S	0.95	0.02
			G724S,T790M	0.88	0.08
			G724S	0.3	0.3
			S768I,V769L	0.8	0.1
			L718V,L858R	0.84	0.06
			G719A,L861Q	0.6	0.3
			L861Q	0.5	0.3
			T790M,V843I,L858R	0.3	0.3

If the Modi–Dunbrack model focuses on features in or near the DFG motif to classify protein conformations as active or inactive, why did our model not use similar features to classify mutants as benign or oncogenic? From Table 2, using the Modi–Dunbrack criteria gave a modest AUC of 0.59. One possibility is that the criteria used by the Modi–Dunbrack model did not vary sufficiently during our simulations to provide useful information for mutant classification. When we examined the variables used by the Modi–Dunbrack model, they did not change much for the proteins during the triplicates of 1-μs simulations. With only a few exceptions, the χ_1_ angle of the phenylalanine remained in the DFG-in active conformation for the proteins (Figure S9). The threonine just in front and the aspartate and phenylalanine of the DFG motif remained in the regions of the Ramachandran map that characterized active conformations (Figures S10 and S11). This does not mean that the Modi–Dunbrack model is not useful for distinguishing between oncogenic and benign proteins; it might just mean that molecular dynamics simulations lasting only a total of 3 μs for each protein were not long enough to observe the conformational switch of the DFG motif. Instead, the machine-learning models found the P-loop and the activation loop to be more useful in distinguishing the proteins with the simulations performed. The simulations did not need to be long enough to obtain “converged” distributions of the conformations of these loops; they just needed to be long enough to capture features that could distinguish oncogenic from benign proteins.

We also note that while the Modi–Dunbrack model is designed to classify protein *conformations* as active or inactive, our models were trained to classify *mutations* as either benign or oncogenic based on the overall properties of the ensemble of conformations sampled by each mutant kinase domain. A sequence of biological events occurs within and beyond the kinase domain to present benign or oncogenic phenotypes. To transmit a growth signal, the kinase domain must bind to a substrate, phosphorylate it, and release the product. For the wild-type proteins and for some mutants, these are preceded by dimerization. Each of these functions can be affected differently by different mutants. The machine-learning model developed here did not model these and other downstream processes explicitly. Rather, it tried to predict healthy or diseased phenotypes from the properties of the conformational ensemble. Nevertheless, the premise of our machine-learning model was that different conformational distributions gave rise to different enzymatic activities, which in turn influenced downstream biological events to yield different disease phenotypes. There is no strong reason to believe that our model could capture biology well beyond molecular processes in which EGFR is directly involved. The modest performance in using the Modi–Dubrack criteria alone in our model to predict oncogenicity might most likely result from the limited sampling in our simulations, as discussed above. The use of machine learning might also improve the connection between differences in the conformational distribution obtained from molecular dynamics simulations and differences in enzymatic activity in an intelligent way, even without directly simulating every step involved in the enzymatic mechanism.

As the machine-learning models identified residues in the P-loop as useful features for classification, we compared the Ramachandran maps for these residues (Figure S12). The variation of the Ramachandran maps among the proteins was larger than that observed in/near the DFG motif, and the random forest models took advantage of these differences to distinguish the two classes of proteins. Figure 7 shows the behavior of the P-loop in another way in which the variation of the residue helicity along the loop is shown. The benign proteins displayed a smaller helicity than the pathogenic proteins did.

**Figure 7:**
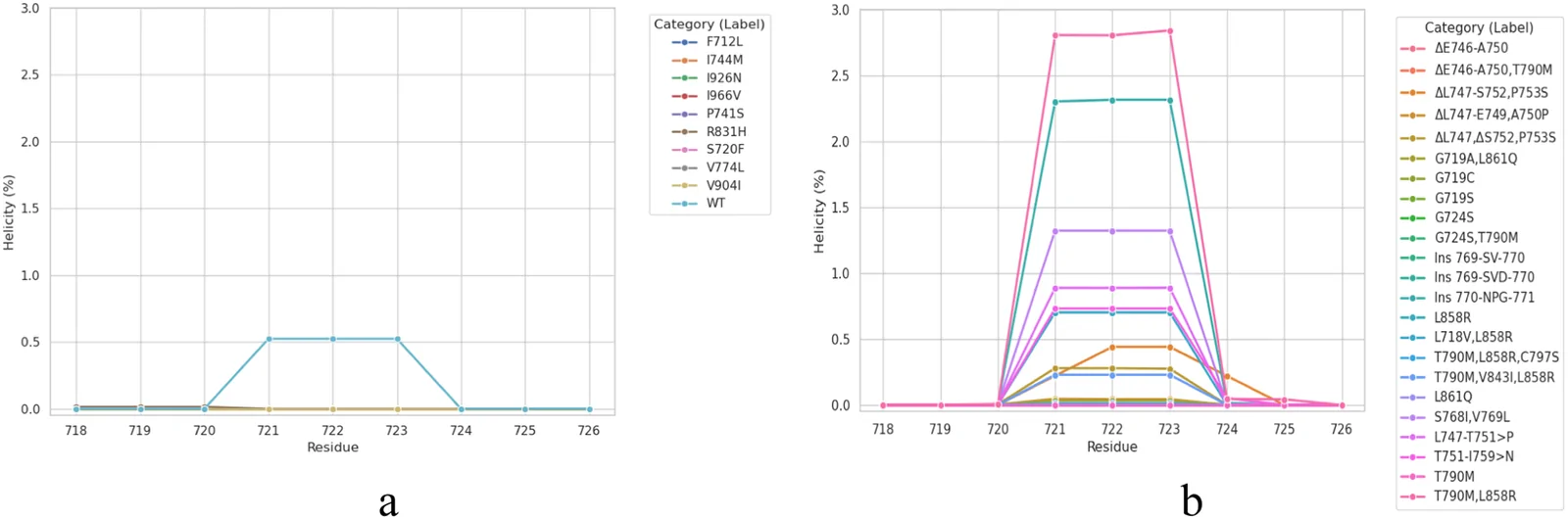
Fractional occupancy of residues in the helical state along the P-loop. (a) Benign proteins and (b) oncogenic proteins.

The activation loop is believed to play an important role in distinguishing between active and inactive proteins. When the activation loop closes, it prevents the binding of substrates, leading to inactivity. In the machine-learning models, we did not explicitly use parameters characterizing the closed or open form of the loop. The models were able to learn this phenomenon indirectly through features such as the propensity of residues forming secondary structure. Figure 8 gives an example of the fractional occupancy of residues in the helical state along the activation loop. A noticeable difference was the helical propensity in two regions along the loop: residues 855–859 and residues 862–867. The helical propensity along residues 855–859 was smaller for the benign proteins than the oncogenic proteins, whereas the reverse was true along residues 862–869. The machine-learning models took advantage of these differences to distinguish the two classes of proteins. The models needed to learn in a more complex way because they were not given features directly reflecting the opening and closing of the activation loop, but they could do so through the complicated architecture of the machine-learning models.

**Figure 8:**
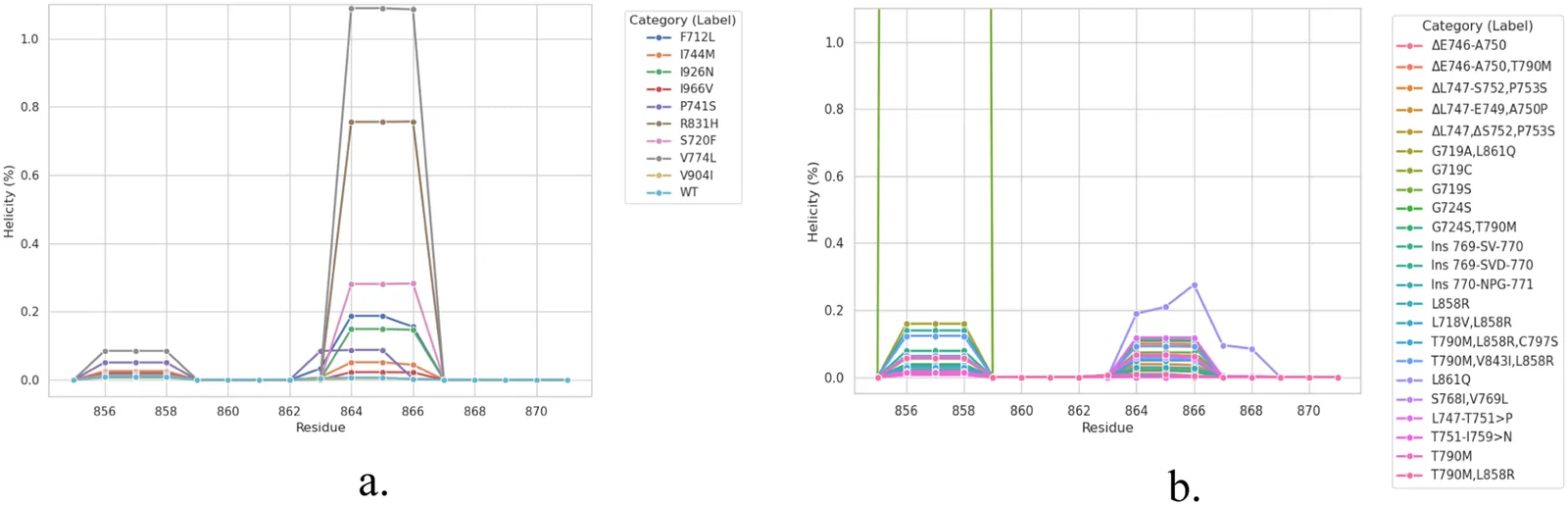
Fractional occupancy of residues in the helical state along the activation loop. (a) Benign proteins and (b) oncogenic proteins. The G719S mutant gave a much larger helical propensity than the other mutants in the region around residues 855–859. It was allowed to go off the scale so that the helical propensities of the other proteins could be compared more easily.

We also examined whether mutations could trigger oncogenicity by enhancing dimerization. Figure 9 shows the solvent-accessible surface area (SASA) of the N-terminal and the C-terminal lobes at the dimer interface. The SASA of the C-terminal region was larger for oncogenic mutants in comparison to benign mutants. The differences for the N-terminal lobe were not as large. It is possible that some of the oncogenic proteins simulated in this study presented larger exposed surfaces to enhance dimerization. On the other hand, some oncogenic proteins could enhance signaling without dimerization. However, the error bars for the SASA were large in the 3-μs simulations for each system (Tables S2 and S3); it is better to be prudent in drawing conclusions until longer simulations and more thorough analyses have been performed.

**Figure 9:**
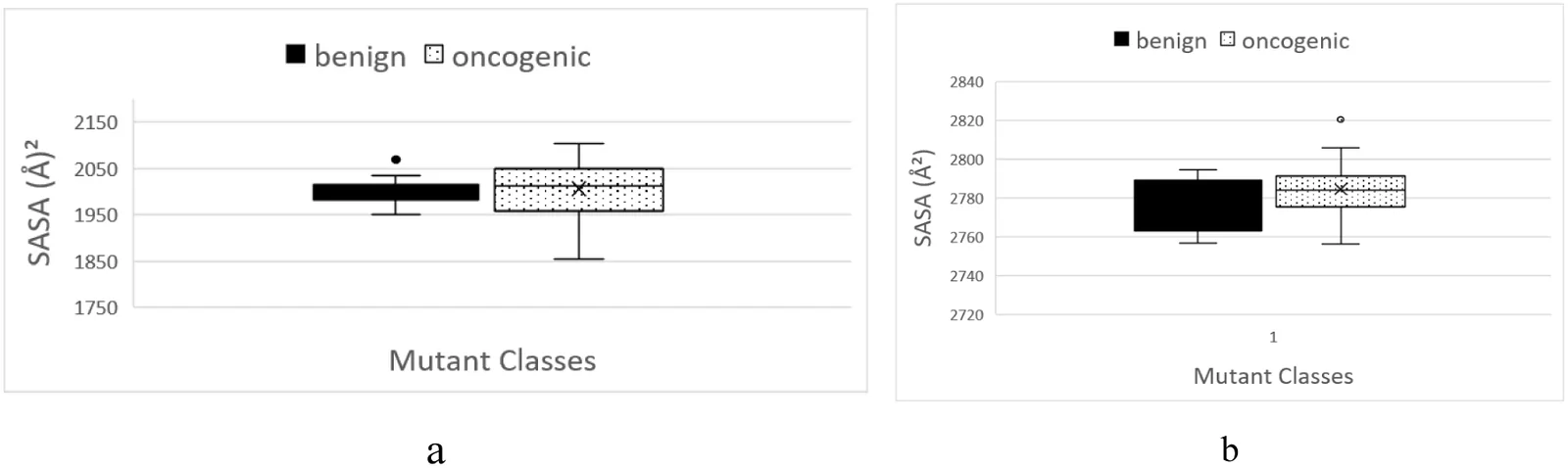
Solvent-accessible surface area (SASA) at the dimer interface. (a) N-terminal lobe. (b) C-terminal lobe.

## Conclusions

We have tested the hypothesis that different conformational distributions of different mutants of EGFR could help distinguish between oncogenic and benign proteins found in lung-cancer patients. We first tested several features extracted from molecular dynamics simulations and found that it was difficult to use these features with single-cutoff values to achieve this goal. However, using some of these features in a machine-learning model (random forest) gave good results, as the model could use these features more intelligently to distinguish the two classes of proteins. Among the features tested, we found that the fraction of time a protein stayed close to the active structure and residue contacts formed gave the best performance when used individually, with AUC values reaching 0.8. Combining these features did not give models better AUC values. Nevertheless, identifying the most important features in the models with good AUC values provided useful insights into factors that distinguish oncogenic from benign proteins.

Although we did not supply features of structural motifs that scientists commonly use to characterize the active and inactive conformations of the kinase domain, the machine-learning models identified some of these structural motifs, such as the P-loop and the A-loop, to be useful for distinguishing active from inactive proteins. On the other hand, the machine-learning models relied less on features such as the DFG motif and the C-helix. This was not because these features were not useful for distinguishing active structures from inactive ones; it was because the molecular dynamics simulations with limited length did not sample the conformational transition between the active and inactive forms of these motifs well, and the machine-learning model did not depend on them to distinguish oncogenic from benign proteins. The machine-learning model did not need to use all common structural motifs for classifying these proteins but only sufficient dynamic structural features to do so.

One might depict this scenario by using the energy landscape theory of protein conformations. The energy landscape of proteins is complex. It is useful to illustrate with a schematic figure like Figure 2 of Ansari et al.^70^ The figure (Figure 10) is more idealized than those that came afterward but can illustrate our findings in a concise manner. The idea was developed by Hans Frauenfelder and coworkers to explain results from their experiments on the photodissociation of small ligands such as CO from myoglobin. They proposed that the energy landscape of a protein forms a hierarchy. The top curve in the figure looks like a single simple energy well, representing the native state of the protein. However, if one amplifies it, one can see at the level below many subwells corresponding to multiple conformational substates of the protein, separating by energy barriers. If one picks a subwell and amplifies it, one can see yet another level of subwells separating by lower energy barriers than the previous level. This hierarchy is continued. The situation in this study is like sampling at a specific level, say, level 3. It contains many subbasins. Each basin represents a conformational substate with different structures adopted by the A loop and the P loop in our simulations. Our simulations did not sample other substrates at higher levels separated by higher energy barriers. Some substrates at the higher level might correspond to states such as having the DFG motif flipped. Our machine-learning model did not need to sample these states at higher levels to distinguish the two classes of proteins; it could utilize conformational substates within level 3 to distinguish the proteins. Yet, it is possible that longer simulations that could sample states separated by higher energy barriers at higher levels could improve the performance of the machine-learning models further. However, the current paper showed that even sampling at the current level could yield useful predictive machine-learning models.

**Figure 10:**
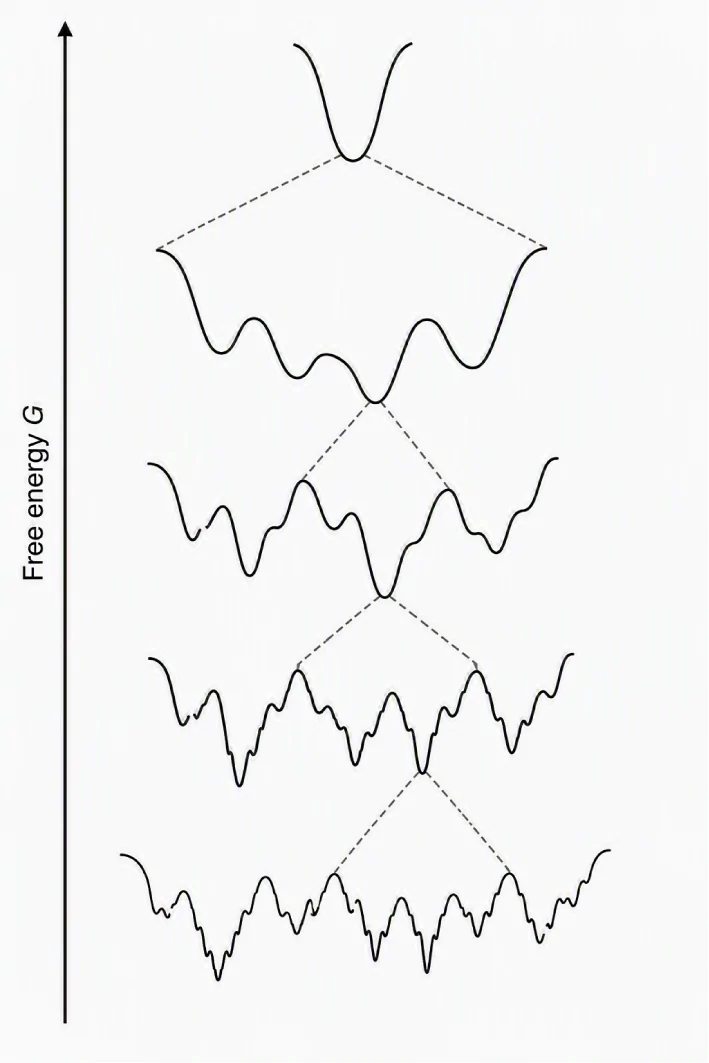
Hierarchical energy landscape of protein conformations following Figure 2 of Ansari et al.^70^

Computational predictions are only considered as Supporting information by the standard operating procedure (SOP) of the Clinical Genome Consortium/Cancer Genome Consortium/Variant Interpretation of Cancer Consortium (ClinGen/CGC/VICC)^14^ for classifying mutants into oncogenic or benign. Predictions from multiple computational programs are only considered as one piece of Supporting information to avoid double counting as many computational programs use similar concepts. However, the approach employed here differs from that of the previous study by explicitly accounting for the conformational distribution of different mutants. This might make it different from other computational approaches and be considered as additional supporting evidence for mutant classification.

For germline mutations, the American College of Medical Genetics and Genomics/Association of Molecular Pathologists (ACMG/AMP)^71^ has long considered computational tools as one way of providing evidence, initially only as supporting evidence (among supporting, modest, strong, and very strong evidence). However, several newer computational tools have been rated to provide moderate or strong evidence recently. It will be interesting to see whether molecular-dynamics-based approaches could add a tool to provide stronger evidence for somatic mutations. This is particularly useful for newly discovered mutants for which not much functional or clinical data are available yet, and a decision needs to be made quickly, such as for cancer patients diagnosed at a late stage. Late-stage patients diagnosed with non-small cell lung cancer have a mean survival rate of only 7.15 months without treatment.^72^ With brain metastasis, the median survival rate reduced further to 1–3 months.^73^ Nearly half of lung cancer patients in the United States were diagnosed at a late stage during 2018–2022 ^74^ because few or no symptoms appear in the early stages. Therefore, when no strong supporting evidence is available yet, computational predictions and other weaker types of evidence might make it possible to identify targeted therapies that might benefit late-stage patients. When available, targeted therapy is more tolerable and effective than chemotherapy.^75^

## Supplementary Material



## Data Availability

Upon acceptance, data, including molecular dynamics simulation trajectories, will be shared via GitHub, Zenodo, and MDRepo.

## Notes

Figures showing time variation of RMSD from the initial structure, the radius of gyration, α-helical content, and β-content; figures showing root-mean-square fluctuations; figure of hierarchical clustering of 33 proteins based on the conformational densities projected onto the first two principal components using the Jensen–Shannon divergence to compute the similarities between two proteins; receiver operating characteristic curves; polar plots showing the χ_1_ angle of the threonine just before the DFG motif and of the aspartate and phenylalanine in the DFG motif; Ramachandran plots of THR 854 and the residues in the DFG motif; Ramachandran plots for residues 718 through 725 in the P-loop; tables showing averages and error bars of root-mean-square deviation (RMSD) from the initial structure averaged over the last 100 ns, fraction of time RMSD within 2 Å of the initial structure, the radius of gyration, and α-helical and β content; tables of solvent-accessible surface area at the dimer interface for the N-terminal and C-terminal lobes (PDF)
