# Supplementary material for: Predicting Oncogenic Mutants of Epidermal Growth Factor Receptor in Lung Cancer by Molecular Dynamics Simulation with Machine Learning
Source: ACS Omega. 2026 Sep 10;11(37):56609–22. doi: 10.1021/acsomega.6c08155 (PMC13613388; doi:10.1021/acsomega.6c08155)
Supplement: Supplementary file 1 [file ao6c08155_si_001.pdf]

Supplementary Information

Predicting Oncogenic Mutants of Epidermal Growth Factor Receptor in Lung Cancer by Molecular Dynamics Simulation with Machine Learning

Victor O. Anyanwu[#], Justin Spiriti[#], and Chung F. Wong*

Department of Chemistry and Biochemistry, University of Missouri-St Louis, 1 University Boulevard, St. Louis, MO 63121

# V.O.A. and J.M.S. contributed equally to this work

*Corresponding Author: Chung F. Wong, email: wongch@umsl.edu

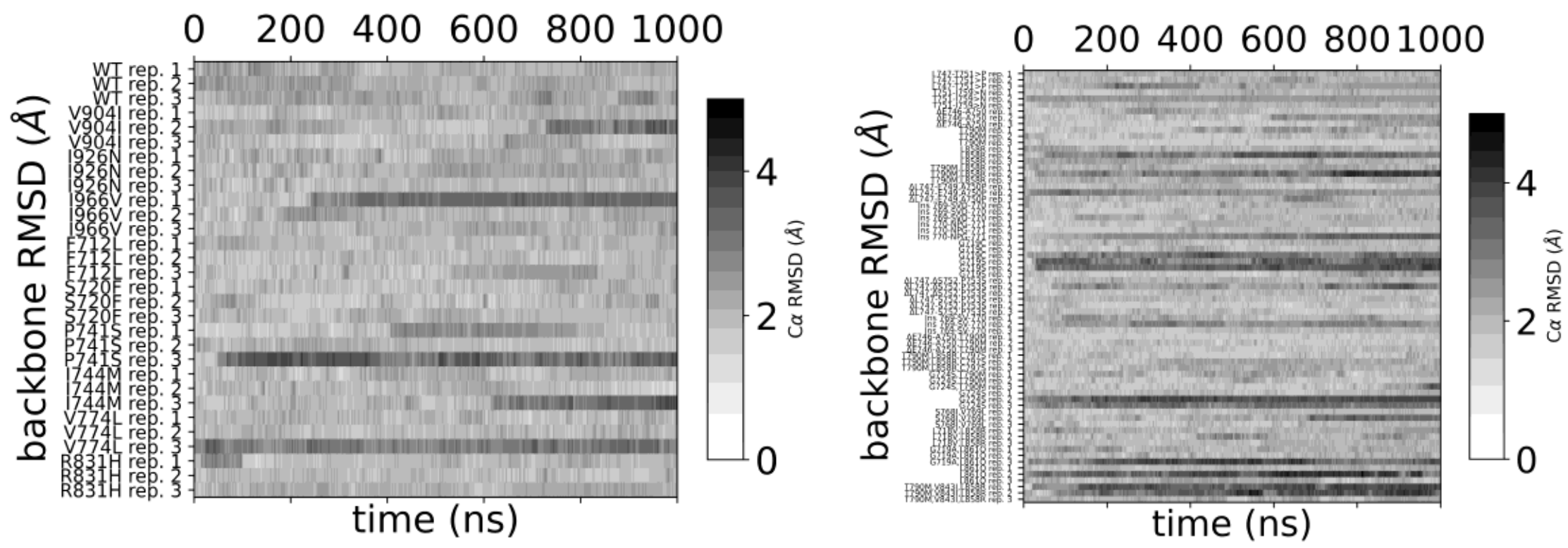

a b

Figure S1. RMSD from initial structure over time, a) benign, b) oncogenic.

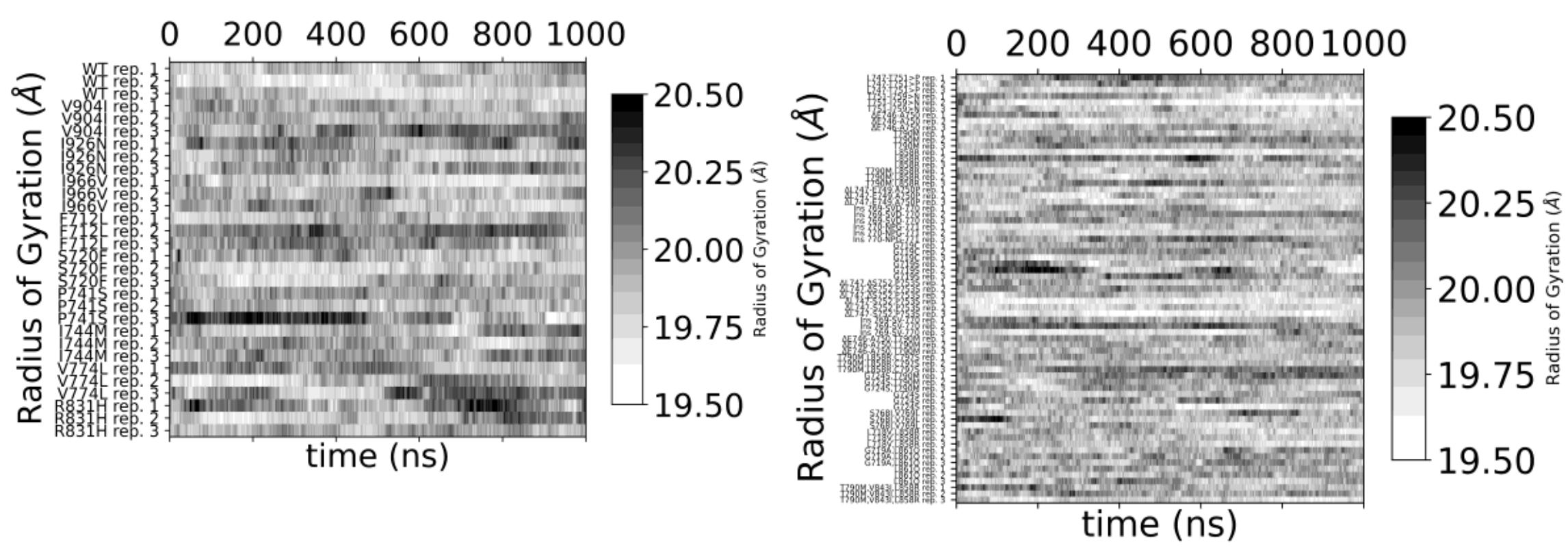

a b

Figure S2: Time course of radius of gyration. a). benign proteins, b). oncogenic proteins.

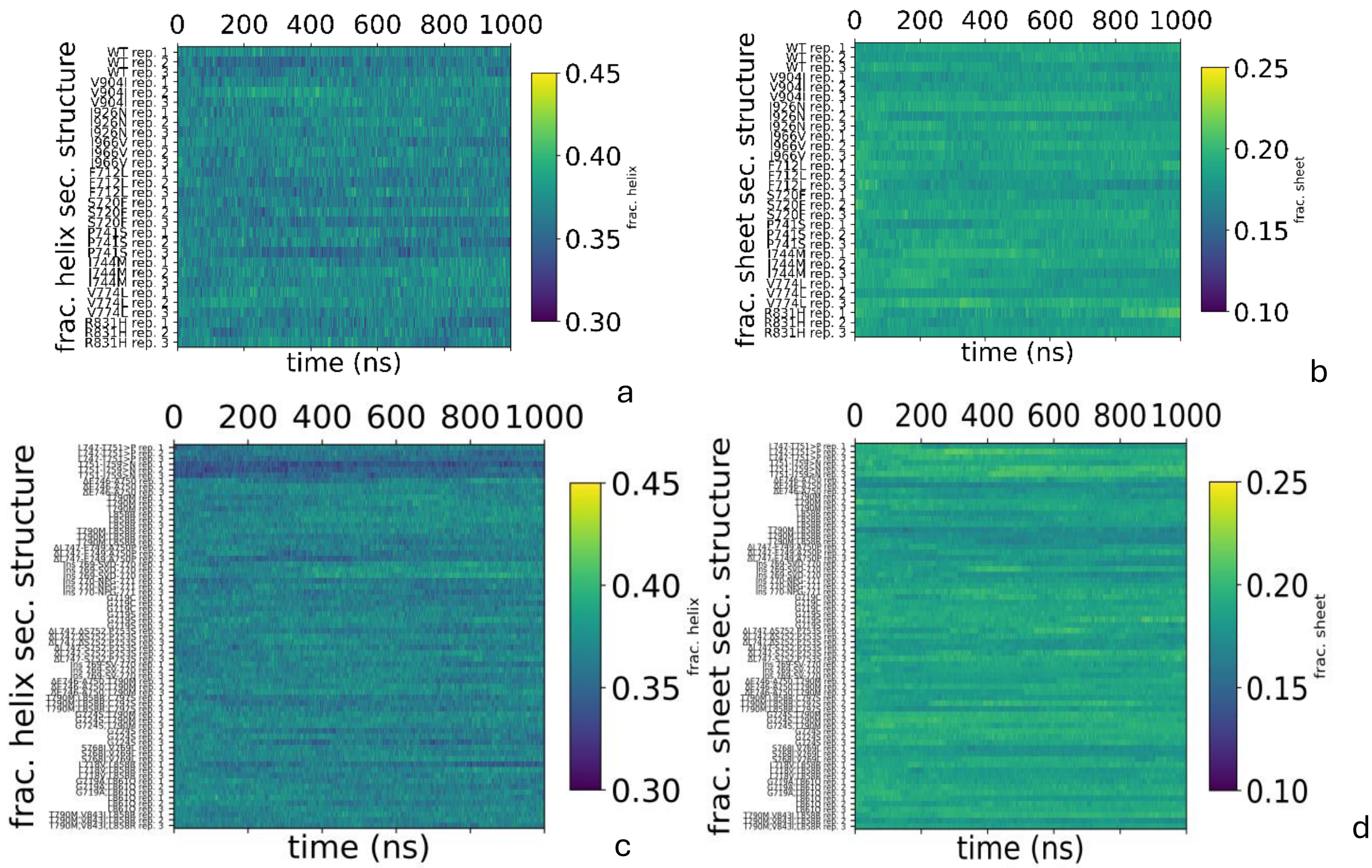

Figure S3: Time course of the fraction of alpha helical and beta structures for each protein simulated. (a) benign/helix, (b) benign/beta sheet, (c) oncogenic/helix, (d) oncogenic/beta sheet.

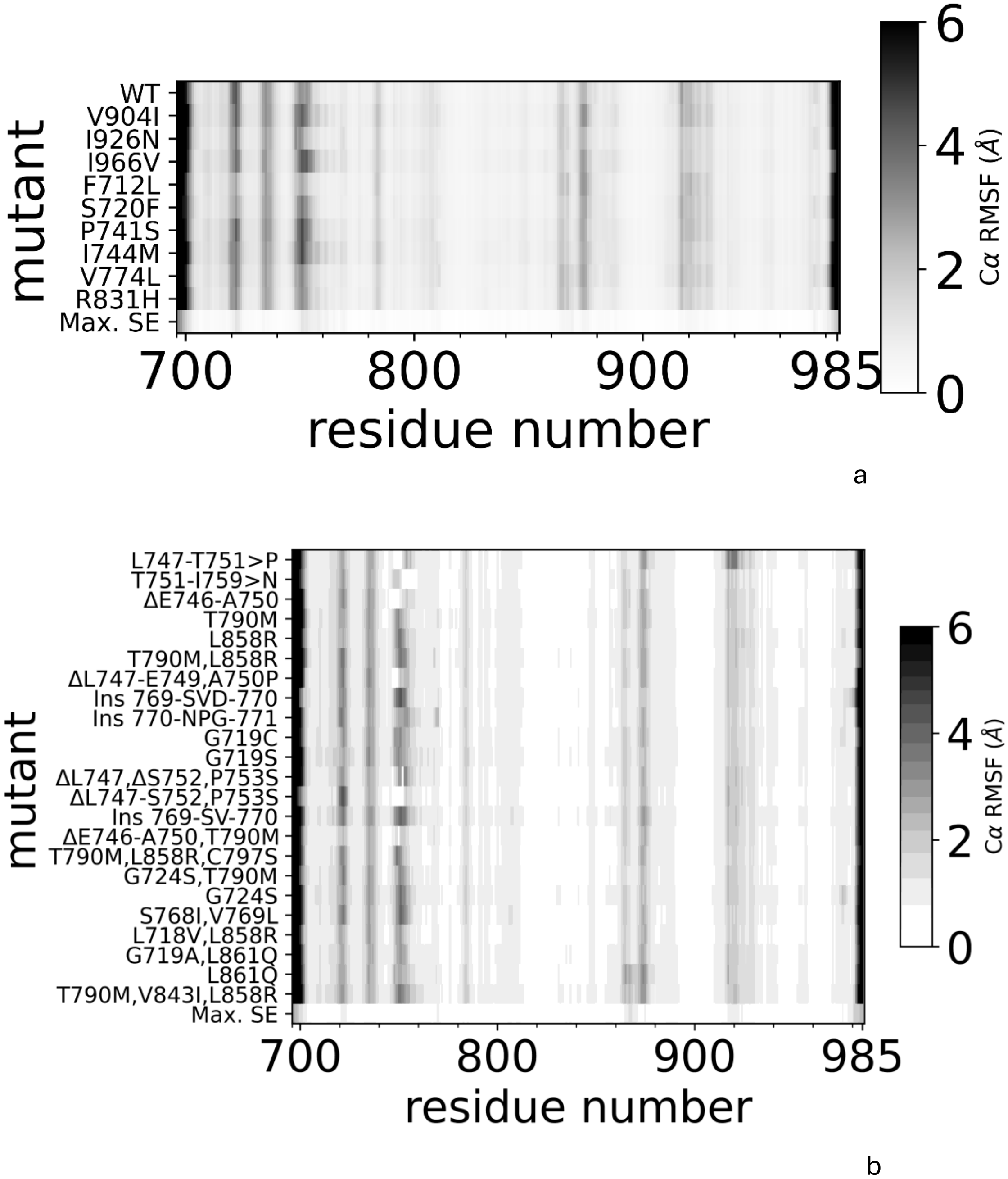

Figure S4: Root-mean-square fluctuations for the proteins studied. (a) benign proteins, (b) oncogenic proteins.

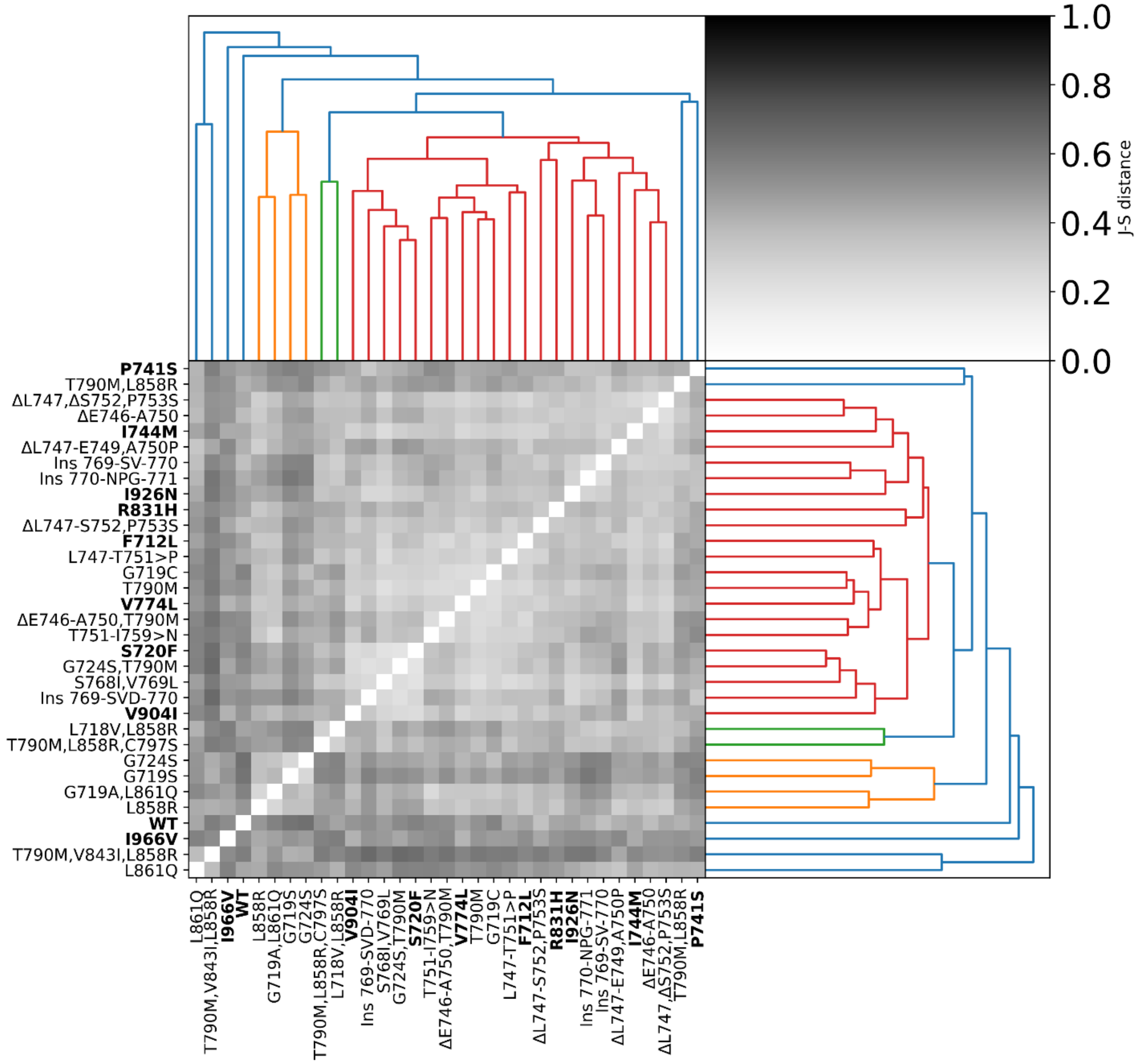

Figure S5: Hierarchical clustering of proteins using population densities projected onto the first two principal components. The similarity between two proteins was calculated by the Jensen-Shannon divergence.

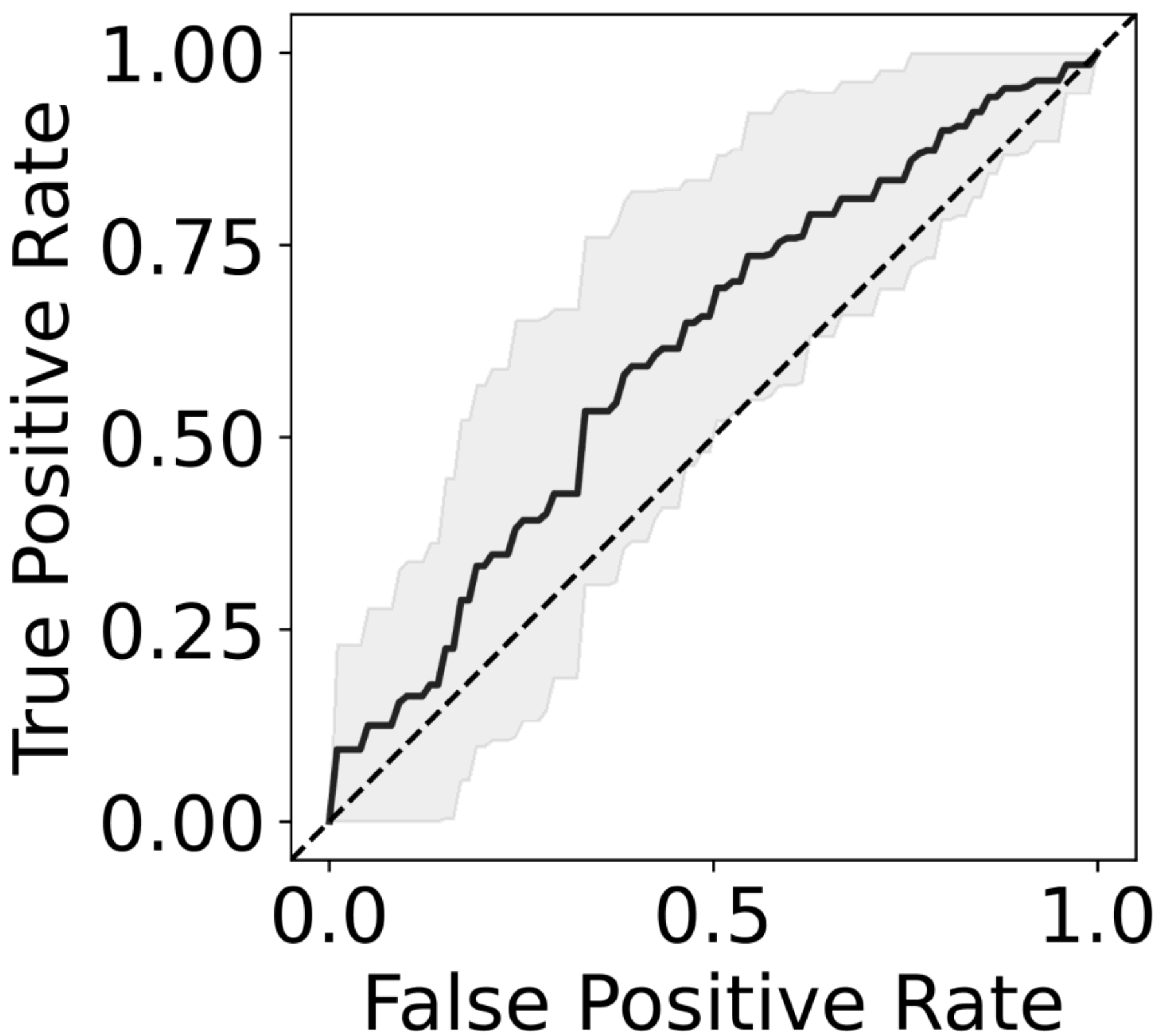

Figure S6: Receiver operating characteristic curve of random forest model using population densities projected onto the first two principal component as features.

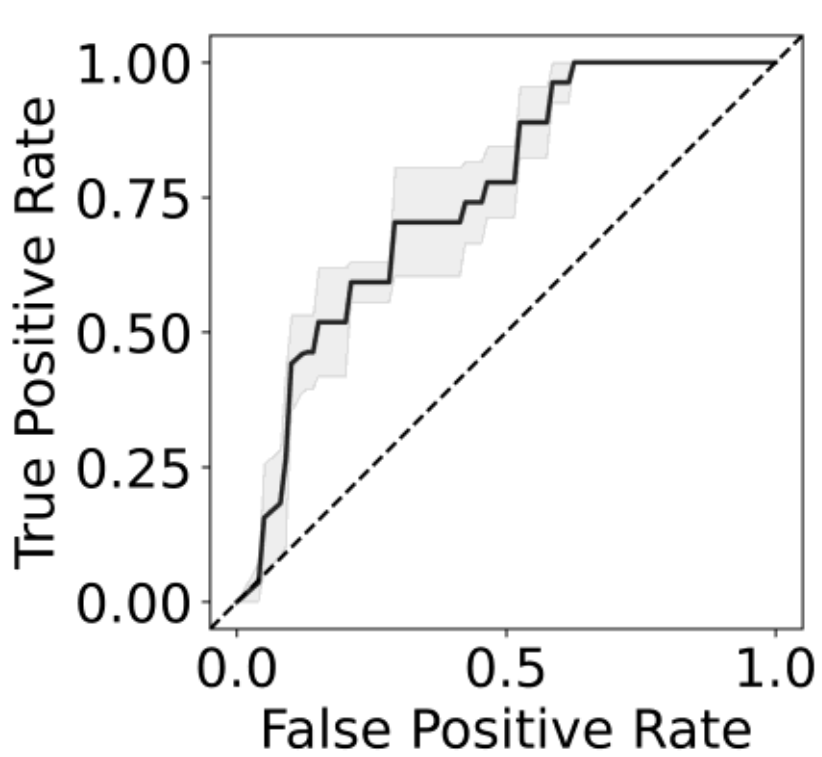

a

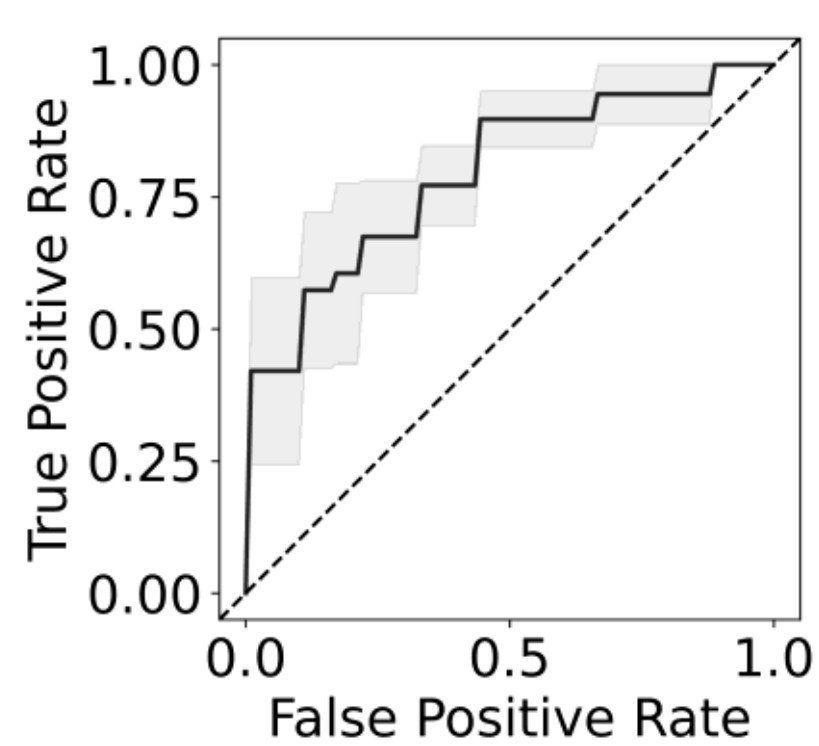

b

Figure S7: Receiver operating characteristic curve of random forest model (a) using as features fraction of time in active conformation and fraction of time structures within 2 Å of the initial structure. (b) using as features fraction of time in active conformation, fraction of

time structures within 2 Å of the initial structure, and fraction of time in alpha helical or beta structure.

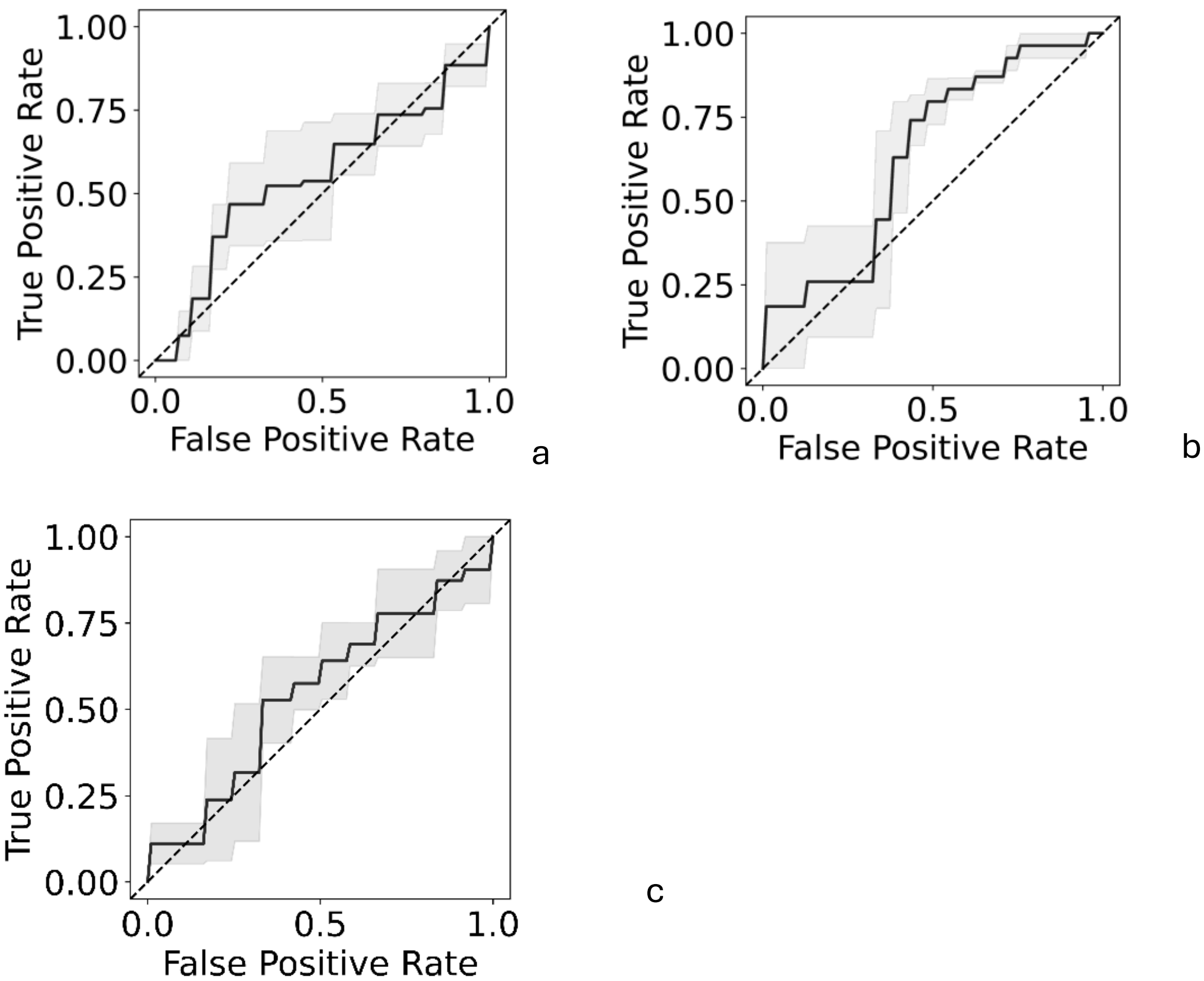

Figure S8: Receiver operating characteristic curve of random forest model (a) using as features fraction of time in active conformation and fraction of time residue contacts formed, (b) using as features fraction of time in active conformation and fraction of time hydrogen bonds formed, (c) using as features fraction of snapshots within 2 Å of initial active conformation and fraction of time each residue contact formed.

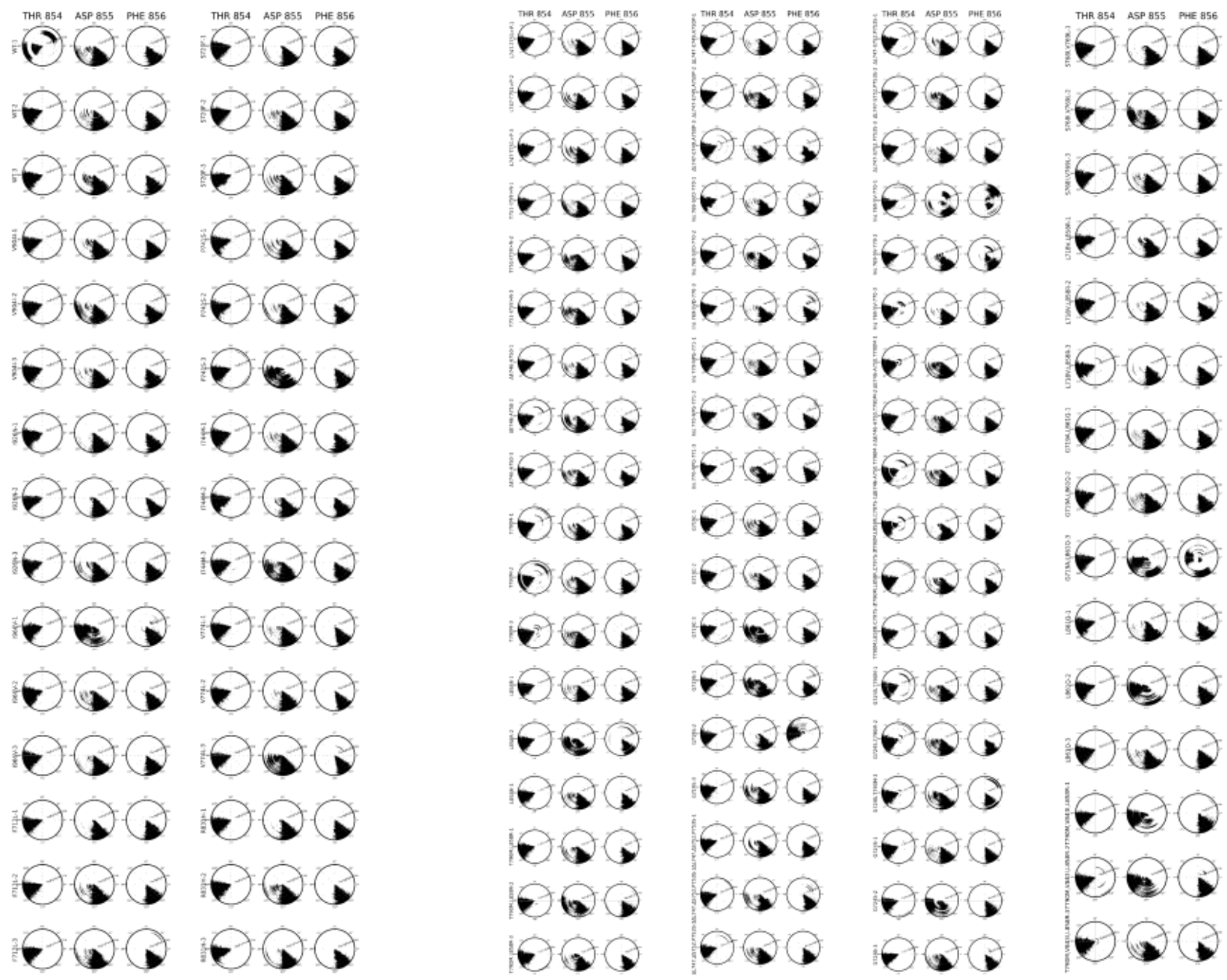

Figure S9: Polar plot of the time course of the χ_1 angle of threonine just before the DFG motif and of the aspartate and phenylalanine in the DFG motif. Left two columns for benign proteins. Right four columns for oncogenic proteins.

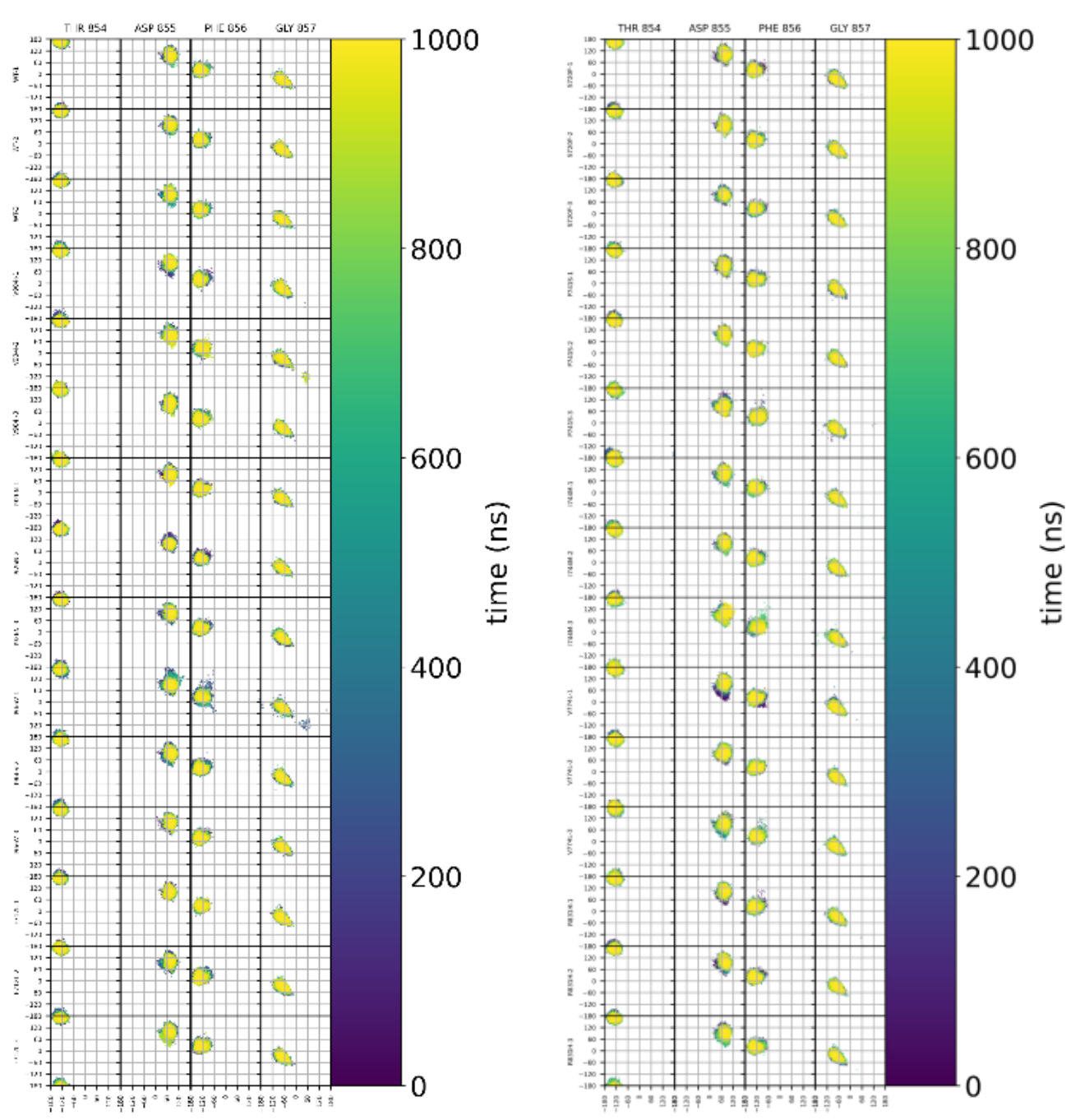

Figure S10: Ramachandran plots of THR 854 and the residues in the DFG motif for benign proteins (colored according to time).

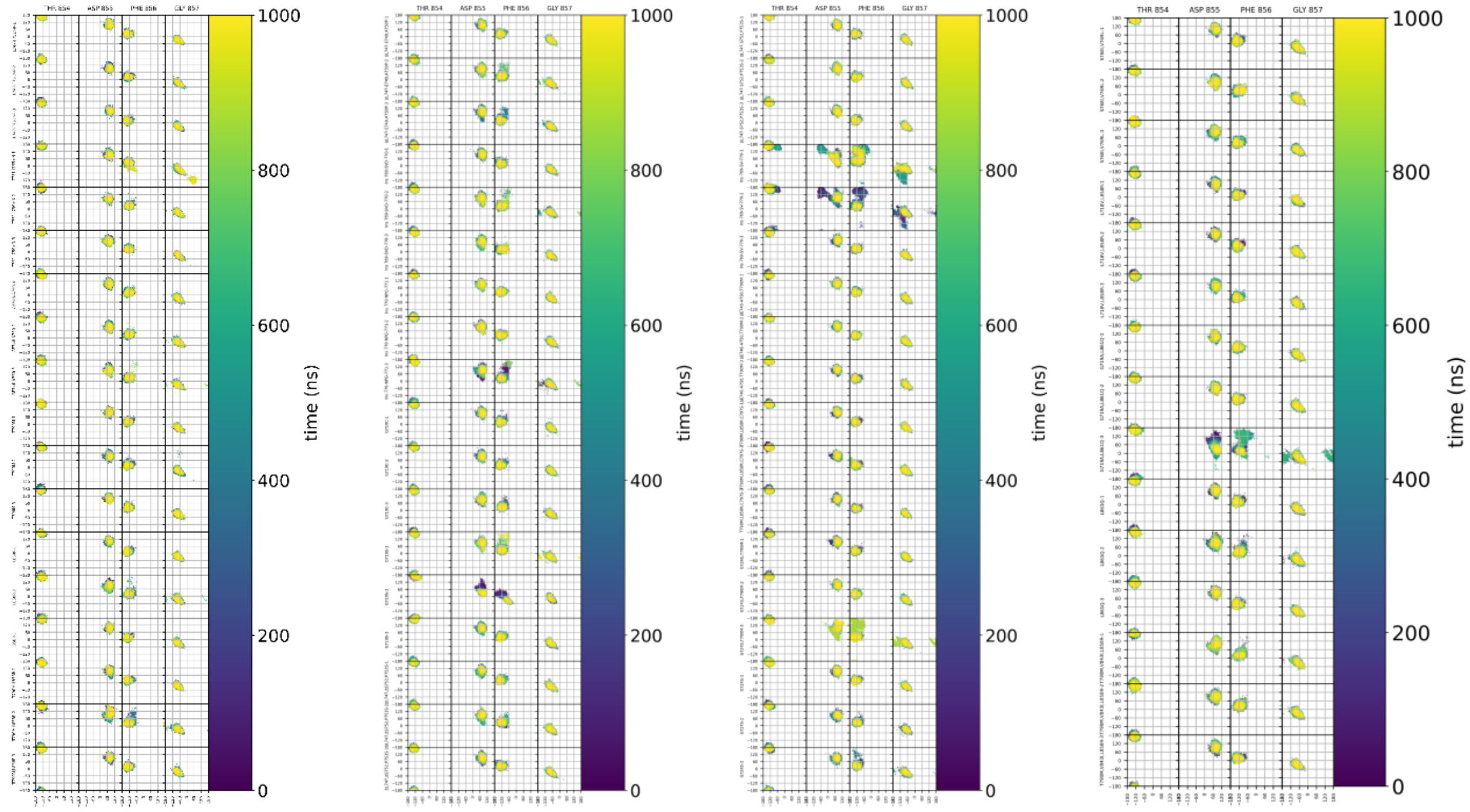

Figure S11: Ramachandran plots of THR 854 and the residues in the DFG motif for oncogenic proteins (colored according to time).

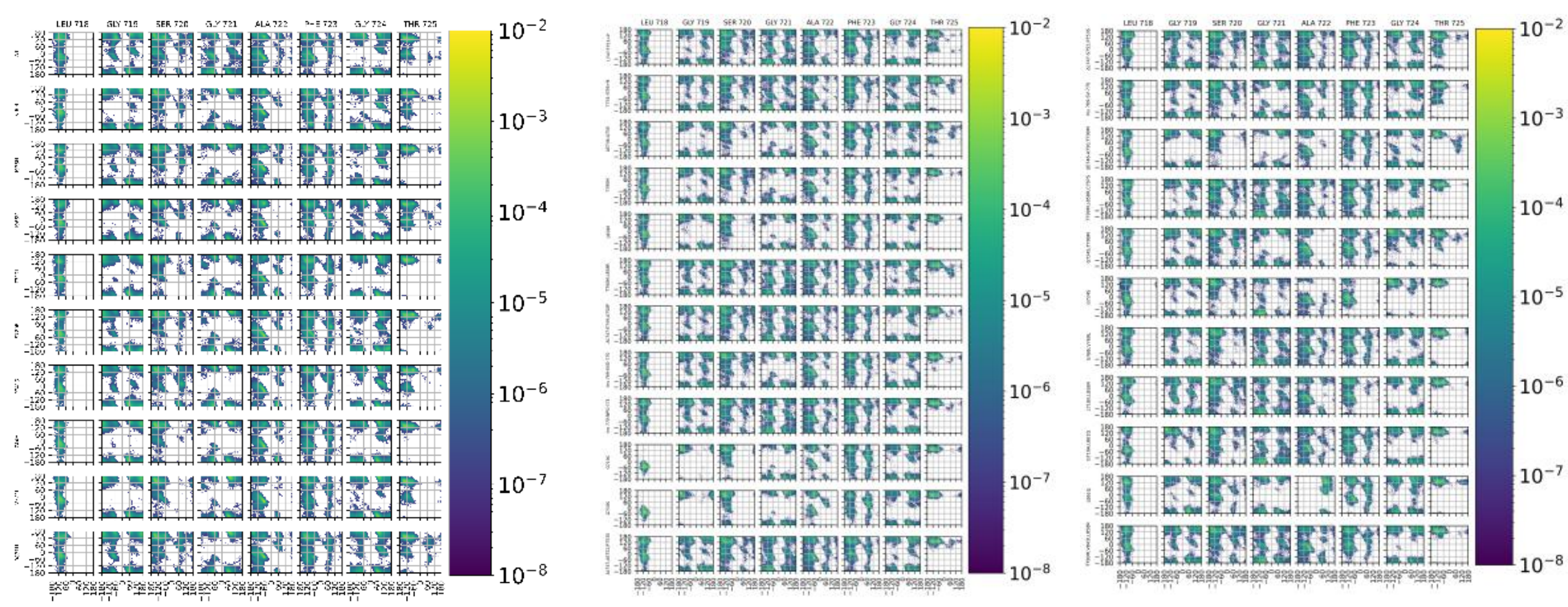

Figure S12: Ramachandran plots for residues 718 through 725 in the P-loop. (a) benign proteins, (b) oncogenic proteins.

Table S1: Average root-mean-square deviation (RMSD) from initial structure, fraction of time RMSD within 2 Å of initial structure, average radius of gyration, and average α- helical and β content.

Protein*	Fraction RMSD within 2 Å	Mean RMSD from last 100 ns	Mean radius of gyration	Mean β structure	Mean α structure
**Wildtype**	0.21±0.02	2.3±0.2	19.88±0.04	17.8±0.1	36.4±0.3
L747-T751>P	0.25±0.07	2.3±0.1	19.88±0.01	18.4±0.4	35.7±0.3
T751-I759>N	0.3±0.2	2.2±0.1	19.7±0.1	19.3±0.2	35.2±0.2
ΔE746-A750	0.58±0.09	2.3±0.2	19.83±0.03	18.6±0.1	36.3±0.2
T790M	0.7±0.1	1.9±0.1	20.00±0.06	18.6±0.1	36.6±0.3
L858R	0.2±0.1	2.3±0.4	19.87±0.03	18.5±0.2	36.3±0.1
T790ML858R	0.18±0.08	2.7±0.6	19.90±0.01	17.9±0.5	36.2±0.5
ΔL747-E749,A750P	0.3±0.2	2.1±0.2	19.89±0.01	17.9±0.2	36.2±0.3
Ins 769SVD770	0.53±0.09	2.08±0.09	19.99±0.03	18.2±0.3	37. 0 ±0.2
Ins 770NPG771	0.5±0.2	2.4±0.5	19.89±0.03	18.2±0.2	35.5±0.2
G719C	0.4±0.2	2.3±0.3	19.88±0.02	18.8±0.1	36.6±0.3
G719S	0.2±0.2	3.3±0.4	19.8±0.1	18.8±0.2	36.5±0.4
ΔL747ΔS752,P753S	0.4±0.2	2.4±0.4	19.85±0.05	18.6±0.1	36.4±0.4
ΔL747-S752,P753S	0.68±0.07	1.87±0.09	19.77±0.05	18.7±0.1	36.6±0.2
Ins 769-SV-770	0.3±0.1	2.2±0.2	19.96±0.05	17.9±0.3	36.2±0.2
ΔE746-A750, T790M	0.83±0.06	1.87±0.06	19.90±0.02	18.7±0.1	36.9±0.2
**V904I**	0.44±0.07	2.4±0.4	19.95±0.05	18.6±0.3	36.5±0.02
**I926N**	0.37±0.09	2.17±0.08	19.89±0.02	17.9±0.1	36.8±0.2
**I966V**	0.29±0.06	2.6±0.4	19.81±0.05	18.7±0.7	36.3±0.4
T790M,L858R,C797S	0.4±0.2	2.09±0.06	19.81±0.05	18.1±0.3	36.8±0.1
G724S,T790M	0.6±0.1	2.3±0.3	19.94±0.03	18.9±0.2	36.8±0.1
G724S	0.2±0.2	3.0±0.5	19.83±0.08	19.3±0.1	35.9±0.3
S768I,V769L	0.5±0.1	2.4±0.4	19.96±0.03	18.4±0.3	35.6±0.4
L718V,L858R	0.39±0.06	2.3±0.1	19.83±0.03	18.1±0.4	36.7±0.2
G719A,L861Q	0.3±0.1	2.6±0.4	19.90±0.08	18.7±0.1	36.3±0.1
L861Q	0.2±0.1	2.5±0.4	19.89±0.02	17.5±0.3	36.4±0.1
T790M,V843I,L858R	0.1±0.1	3.4±0.5	19.74±0.04	18.5±0.2	35.7±0.8
**F712L**	0.54±0.06	2.06±0.03	19.88±0.04	18.3±0.3	36.5±0.1
**S720F**	0.56±0.04	2.08±0.05	19.9±0.01	18.2±0.3	36.2±0.3
**P741S**	0.4±0.2	2.4±0.5	19.88±0.03	18.2±0.3	36. 0 ± 0.2
**I744M**	0.44±0.06	2.4±0.6	19.94±0.01	18. 0 ±0.5	36.5±0.2
**V774L**	0.3±0.2	2.4±0.3	19.92±0.03	18.2±0.4	36.4±0.2
**R831H**	0.4±0.1	2.0±0.1	19.87±0.06	18. 0 ±0.4	36.8±0.2

*Note that only the average RMSD from initial structure was calculated using the last 100 ns of each trajectory. All other averages were computed with the whole trajectories.

Table S2: Solvent-accessible surface area at the dimer interface for the N-terminal and C-terminal lobes for the benign proteins

Protein	SASA (N-terminal) (Å^2)	SASA (C-Terminal) (Å^2)
Wildtype	1980±50	2760±10
V904I	2069±4	2760±20
I926N	1990±40	2790±10

I966V	2000±40	2780±20
F712L	1980±10	2795±3
S720F	1950±30	2790±10
P741S	1990±30	2770±1
I744M	2040±30	2780±5
V774L	1980±20	2760±10
R831H	1980±30	2787±9

Table S3: Solvent-accessible surface area at the dimer interface for the N-terminal and C-terminal lobes for the oncogenic proteins

Mutants	SASA (N-terminal) ($Å^2$)	SASA (C-terminal) ($Å^2$)
L747-T751>P	2103±30	2780±20
T751-I759>N	1850±30	2790±10
ΔE746-A750	1970±20	2780±10
T790M	2060±20	2780±8
L858R	2010±30	2780±30
T790ML858R	2070±50	2790±10
ΔL747-E749,A750P	2070±60	2760±10
Ins 769SVD770	1960±40	2769±3
Ins 770NPG771	1950±80	2820±40
G719C	1960±10	2800±40
G719S	1990±10	2773±6
ΔL747ΔS752,P753S	2040±50	2786±7
ΔL747-S752,P753S	2030±60	2780±10
Ins 769-SV-770	2050±60	2792±3
ΔE746-A750, T790M	2020±20	2776±8
T790M,L858R,C797S	2000±10	2800±20
G724S,T790M	1930±50	2790±10
G724S	1960±50	2800±30
S768I,V769L	2080±10	2760±30

L718V,L858R	2020±20	2788±9
G719A,L861Q	2000 ±40	2770±10
L861Q	1990±30	2784±4
T790M,V843I,L858R	2010±30	2810±10
